# Associations of ADHD genetic risk with objective and subjective economic wellbeing: evidence from Finnish population-based cohorts

**DOI:** 10.1007/s00127-026-03183-4

**Published:** 2026-09-01

**Authors:** Aaro Hazak, Johanna Liuhanen, Katri Kantojärvi, Sonja Sulkava, Veikko Salomaa, Seppo Koskinen, Markus Perola, Tiina Paunio

**Affiliations:** 1 Finnish Institute for Health and Welfare, Department of Public Health, Helsinki, Finland; 2 Department of Psychiatry/SleepWell Research Program, Faculty of Medicine, University of Helsinki, Helsinki, Finland; 3 Department of Finance, Aalto University, Espoo, Finland; 4 Department of Economics and Finance, Tallinn University of Technology, Tallinn, Estonia; 5 Department of Psychiatry, HUS Helsinki University Hospital, Helsinki, Finland; 6 Department of Clinical Genetics, HUS Helsinki University Hospital, Helsinki, Finland; 7 Department of Internal Medicine, University of Turku, Turku, Finland; 8 Research Program for Clinical and Molecular Metabolism, Faculty of Medicine, University of Helsinki, Helsinki, Finland

**Keywords:** Neurodevelopmental diversity, Polygenic risk, Educational attainment, Occupational outcomes, Income, Economic satisfaction

## Abstract

**Purpose:**

This study examines how genetic liability for Attention-Deficit/Hyperactivity Disorder (ADHD), measured using a polygenic index (ADHD-PGI), relates to both objective and subjective socio-economic outcomes in the general population.

**Methods:**

We studied six Finnish population-based cohorts (1992–2017; *N* = 20,121), linking genetic, registry and survey data. Probit and ordered probit models estimated associations between ADHD-PGI and sequential socio-economic outcomes: educational attainment, employment, occupational selection, income and economic satisfaction.

**Results:**

Higher ADHD-PGI was strongly associated with lower educational attainment (*P* < 0.001); individuals in the top ADHD-PGI quintile were 9.2% points (pp) less likely to complete higher education than those in the bottom quintile. ADHD-PGI was negatively associated with employment (*P* < 0.001; −2.6 pp for top vs. bottom quintile). It predicted lower likelihood of knowledge work and higher likelihood of physical work (both *P* < 0.001), with effects persisting beyond educational differences. ADHD-PGI was associated with lower equivalent income (*P* < 0.005) and remained significant within the higher-education subgroup (*P* = 0.006); however, associations were not significant within specific occupational categories, suggesting that income inequalities primarily reflect educational and occupational sorting rather than within-occupation pay gaps. ADHD-PGI was also linked to lower economic satisfaction (*P* < 0.001), an association that persisted among males in the lowest income tertile (*P* < 0.001), indicating pathways beyond objective income.

**Conclusion:**

Genetic liability for ADHD is associated with economic disadvantage across multiple domains—lower education, reduced employment, occupational segregation and lower income—as well as less favourable subjective economic evaluations. Considering ADHD-related traits may enhance understanding of socio-economic disparities and workforce inclusion in the general population beyond clinically diagnosed ADHD.

**Supplementary Information:**

The online version contains supplementary material available at https://doi.org/10.1007/s00127-026-03183-4.

## Introduction

Attention-deficit/hyperactivity disorder (ADHD) is a neurodevelopmental condition characterised by inattention, impulsivity, and hyperactivity, with an estimated heritability of approximately 70–80% [1–3]. Although ADHD is commonly diagnosed in childhood, its effects often extend into adulthood, associating with cognitive functioning, educational achievement, employment, and income [4–9]. Beyond clinically diagnosed ADHD, associated behavioural and cognitive traits—including impulsivity, emotional dysregulation, executive dysfunction, and attentional difficulties such as distractibility and reduced sustained attention—occur along a continuum in the general population and may contribute to socio-economic disparities [2, 8].

Attention is a limited cognitive resource that increasingly determines success in education and work. Loewenstein and Wojtowicz describe attention as a scarce and valuable commodity in modern economies, where individuals must constantly allocate limited mental focus across competing demands [10]. From this perspective, variation in attentional capacity—such as that associated with ADHD-related traits—can influence human capital formation and labour market performance by shaping how individuals learn, process information, and manage complex work environments. Applying genetic proxies for ADHD to study economic outcomes thus extends the “economics of attention” framework into the epidemiological domain, linking neurodevelopmental variation to long-term socio-economic trajectories.

Advances in genome-wide association studies (GWAS) enable the estimation of individual genetic liability for ADHD through polygenic indices (PGI) [11, 12], creating opportunities to investigate how inherent ADHD-related traits relate to economic outcomes. Prior research has revealed substantial genetic correlations (rg) between ADHD and key socio-economic traits, including fluid intelligence (rg = −0.41), educational attainment (rg = −0.54), employment (rg = −0.28), income (rg = −0.50), and socio-economic status (rg = −0.66) [12–15]. These correlations suggest that ADHD-related genetic risks may be linked to economic outcomes through multiple pathways, such as reduced educational attainment, cognitive functioning, occupational sorting, and workplace performance. Individuals with elevated ADHD polygenic risk may face challenges in structured educational environments, leading to lower formal qualifications and fewer opportunities for high-income careers [16]. Additionally, impulsivity and risk-taking tendencies associated with ADHD may shape occupational choices, self-employment patterns, and financial decision-making [17].

While earlier studies have linked ADHD polygenic risk to lower education, income, and employment [16, 18, 19], less is known about how these relationships unfold sequentially—how earlier life outcomes, such as education and consequent occupational sorting, may shape later economic wellbeing. In addition, most research has focused exclusively on objective socio-economic indicators. However, socio-economic wellbeing also has an important subjective dimension reflecting individuals’ perceived adequacy of resources and satisfaction with their financial situation. Examining both dimensions is analytically informative because objective outcomes capture material resources, whereas subjective evaluations reflect how individuals experience and interpret those resources in everyday life. Studying these dimensions jointly can therefore help distinguish whether socio-economic disadvantages associated with ADHD-related traits operate primarily through structural pathways (e.g., education and labour market outcomes) or also involve psychological and behavioural mechanisms affecting individuals’ perceptions of their economic situation.

This study examines associations between ADHD polygenic index and sequential dimensions of economic well-being—educational attainment, employment, occupational segregation, income, and perceived economic satisfaction—using data from over 20,000 Finnish adults observed between 1992 and 2017. Finland’s relatively egalitarian social context, with universal access to education and healthcare, offers a unique setting for investigating how genetic liability translates into socio-economic outcomes under conditions that seek to minimise structural inequality.

Recognising reported gender differences in ADHD presentation [20] and the rising demand for cognitive skills in knowledge-based economies [21], we also examine whether these associations differ between males and females. Although heritability estimates do not suggest major sex differences [22, 23], some average differences in symptom profiles and comorbidities have been reported: men more often exhibit hyperactive–impulsive symptoms, while women more frequently present with inattentive symptoms and emotional dysregulation [24, 25]. Gender differences in psychiatric comorbidities mirror patterns in the general population—men show higher prevalence of substance use and psychotic disorders, while women are more likely to experience anxiety, depression, and bipolar disorder [25, 26]. These differences represent population-level tendencies, and substantial heterogeneity and overlap exist across genders.

Because ADHD-related traits exist along a spectrum, the societal burden of ADHD likely extends beyond diagnosed cases. Individuals who are undiagnosed or subclinically affected may also experience educational and occupational disadvantages, amplifying long-term socio-economic inequality. Using ADHD-PGI as a genetic proxy allows examination of these broader population-level effects while mitigating reverse causality and some forms of confounding. Nonetheless, ADHD-PGI captures only a fraction of the genetic contribution to ADHD and does not account for environmental factors, gene–environment interplay, or rare genetic variation [12, 27]. Although it cannot fully disentangle direct genetic from environmental influences, ADHD-PGI remains a valuable tool for identifying predispositions that lie outside individual control.

Pleiotropy—where genetic variants influence multiple traits—remains an important consideration, as ADHD shares genetic architecture with other psychiatric and somatic disorders, including autism spectrum disorder (ASD; rg = 0.42), schizophrenia (rg = 0.17), major depressive disorder (MDD; rg = 0.31), and cannabis use disorder (rg = 0.61) [12]. Mendelian randomisation studies further suggest potential causal links between ADHD and MDD [28, 29]. A substantial proportion of genetic variants associated with ADHD also contribute to other psychiatric conditions, with over 90% overlapping with schizophrenia and MDD, and approximately 84% with ASD [12]. These and other conditions, such as cardiometabolic diseases, are frequently observed as comorbidities of ADHD [30–32]. Up to 80% of individuals with ADHD have at least one comorbid psychiatric disorder [33], underscoring its broad public health relevance.

By integrating genetic, registry, and survey data, this study contributes new population-based evidence on how genetic liability for ADHD is associated with both objective economic trajectories and subjective experiences of economic well-being. Studying these outcome dimensions jointly allows assessment of whether genetic liability is associated only with structural economic disadvantages—such as lower education, employment, or income—or also with how individuals perceive their economic circumstances when objective resources are comparable. Elucidating these associations can enhance a more comprehensive understanding of the mechanisms linking ADHD genetic liability to socio-economic outcomes and contribute to broader epidemiological research on the social consequences of ADHD genetic risk in the population.

## Methods

### Study population and data sources

This study utilised pooled genetic and survey data from the Finnish National FINRISK Study (FR; 1992–2012) [34] and the FinHealth 2017 Study (FH) [35], merged with national registry data on education, labour market status, and health. These repeated cross-sectional, regional population-representative surveys provide comprehensive information on Finnish adults. Given that genetic predispositions remain stable across the life course and that education and occupational trajectories are largely established in adulthood, the cross-sectional nature of the data does not limit the study’s ability to assess economic outcomes.

Approval for this study was granted by the Finnish Institute for Health and Welfare, with ethical approval from the Coordinating Ethical Committee of the Helsinki and Uusimaa Hospital District (approval numbers: 38/96, 558/E3/2001, 229/E0/06, 162/13/03/00/2011, and 37/13/03/00/2016). All participants provided written informed consent, and the study adhered to the principles of the Declaration of Helsinki.

The final analytic sample included 20,121 individuals aged 25–64 after excluding those with missing genetic or registry data, those from Lapland (due to limited cohort coverage), and households exceeding 10 members or containing more than one additional adult beyond the respondent, ensuring more precise income attribution. The participant flowchart is provided in the Appendix A of Hazak et al. [36].

### Genetic measures

Genotyping was conducted separately for the FH and FR cohorts, with minimal overlap in participants. The ADHD-PGI was derived using summary statistics from a large GWAS on ADHD [11], which included 20,183 cases and 35,191 controls of European ancestry. PRS-CS Bayesian regression was applied to estimate posterior variant effect sizes, using the European 1000 Genomes Project reference panel. The ADHD-PGI for each participant was computed as a weighted sum of risk allele counts, standardised to a mean of 0 and a standard deviation of 1 in the study population. Further details on genotyping and imputation procedures are available in the Appendix A of Hazak et al. [36].

To validate the ADHD-PGI as an indicator of ADHD liability in our sample, we compared the distribution of ADHD-PGI scores between individuals with a recorded ADHD diagnosis in the Finnish Care Register for Health Care (HILMO) and those without such a diagnosis. The HILMO register, maintained by the Finnish Institute for Health and Welfare, covers inpatient medical treatment, and only a small proportion of ADHD cases are captured in this dataset (*N* = 28 diagnosed vs. 20,093 without diagnosis). Because most ADHD cases are treated in outpatient settings and thus classified among the non-diagnosed group, this comparison provides a conservative (lower-bound) estimate of group differences. A Wilcoxon rank-sum test indicated a statistically significant difference in ADHD-PGI between the groups (*P* = 0.003), supporting the score’s validity as a proxy for ADHD liability in our sample. It should be noted that polygenic indices capture common-variant genetic liability on a continuous dimension and typically explain a modest share of variance in complex traits, consistent with the relatively low heritability attributable to common genetic variants reported for ADHD in GWAS.

### Outcome measures

Education: Educational attainment was categorised into three ordered levels—primary, secondary, and higher—based on Statistics Finland registry data, reflecting the highest completed qualification. Primary education corresponds to no qualification beyond compulsory schooling (International Standard Classification of Education, ISCED 2011 levels 0–2). Secondary education includes upper secondary general and vocational education as well as post-secondary non-tertiary qualifications (ISCED 2011 levels 3–4). Higher education comprises short-cycle tertiary, bachelor’s, master’s, and doctoral degrees (ISCED 2011 levels 5–8). This categorisation is overall consistent with standard groupings used in Finnish labour market and inequality research, where basic education is associated with lower employment rates and earnings [37]. Comparisons with annual country-level statistics from Statistics Finland indicate that the distribution of educational levels in our analytic sample broadly reflects national population patterns: the proportion with primary education is modestly lower, and the proportions with secondary and higher education somewhat higher, than national averages. These differences do not exceed 10% points in any year and likely reflect the regional composition of the cohorts and modest participation bias towards individuals with higher education in genotyped samples [38].

Labour Market Status: Labour market status was derived from Statistics Finland’s national administrative registry of socio-economic position, which follows the United Nations Economic Commission for Europe (UNECE) recommendations for population censuses and is broadly comparable to the International Standard Classification of Occupations (ISCO-08). Employment status was defined according to official register-based labour market position at the beginning of each study year (for the FR 1992, 1997, and 2002 cohorts, status referred to the preceding year). Individuals were classified as employed if they were registered as wage earners or self-employed. The non-employment category included all individuals not registered as employed at the relevant time point. This group comprised both the unemployed and persons outside the labour force, including those on parental or caregiving leave, individuals undertaking mandatory military or civil service, recipients of disability-related benefits, students without registered employment, and other persons not participating in the labour market. This definition corresponds to standard register-based labour market classifications used in Finnish population research, and the observed non-employment rate (24.6%; Table 1) reflects the share of adults aged 25–64 who were not registered as employed at a given measurement year. Such non-employment proportions are consistent with national employment and labour market participation patterns in Finland over recent decades, as documented in labour market analyses, e.g. [39]. Among the employed, occupational position was categorised into four mutually exclusive groups based on the national socio-economic classification. Self-employed refers to individuals registered as entrepreneurs or own-account workers. Physical work comprises manual occupations broadly corresponding to ISCO-08 major groups 6–9. Office work includes lower-level administrative and clerical occupations (approximately ISCO-08 major groups 4–5). Knowledge work encompasses upper-level administrative, managerial, professional, and related occupations, broadly consistent with ISCO-08 major groups 1–3. This categorisation reflects widely used distinctions in Finnish labour market and inequality research between manual, clerical, and professional/managerial occupations.

Income: Household income was obtained from survey data in categorical brackets. Because bracket thresholds varied across survey years and currency regimes (FIM prior to 2002; EUR thereafter), we first converted income categories into a semi-continuous measure by assigning the midpoint of each bracket. Income was then equivalised using the OECD-modified equivalence scale (first adult = 1.0; additional adult = 0.5; children = 0.3) to account for household composition [40], yielding comparable estimates of disposable resources per household member. To ensure comparability across study years with differing bracket structures and macroeconomic conditions, equivalent income was subsequently standardised within survey year and categorised into tertiles (lowest, middle, highest). This relative income measure captures individuals’ position within the contemporaneous income distribution rather than absolute monetary levels. The tertile specification facilitates interpretation in ordered probit models and enhances cross-cohort comparability.

Economic Satisfaction: Self-reported satisfaction with personal financial situation was derived from survey data and recoded into a binary variable: satisfied (very satisfied, satisfied, or somewhat satisfied) versus unsatisfied (unsatisfied or very unsatisfied).

### Control measures

All models controlled for age (linear and squared), gender, birth cohort, study year, and three principal components of genetic ancestry to adjust for population stratification. This approach aligns with best practices for genetic studies in Finnish populations, given their relative genetic homogeneity.

### Descriptive statistics

Table 1 presents descriptive statistics, Supplemental Fig. 1 histograms of ADHD-PGI by gender, and Supplemental Table 1 pairwise linear correlation matrix of study variables.

**Table 1:** Descriptive statistics

Variable	Explanation	All		Male		Female		Lowest quintile ADHD-PGI^a^		Highest quintile ADHD-PGI^a^	
		Mean / %	Min Max SD	Mean / %	Min Max SD	Mean / %	Min Max SD	Mean / %	Min Max SD	Mean / %	Min Max SD
N		20,121		9,248		10,873		4,025		3,980	
ADHD-PGI^a^		-0.005	-3.809	-0.017	-3.809	0.005	-3.683	-1.399	-3.809	1.765	0.842
			4.400		3.702		4.400		-0.849		4.400
			0.995		1.004		0.987		0.466		0.466
Education											
1	Primary	20.2%		22.1%		18.5%		17.3%		23.4%	
2	Secondary	42.1%		44.9%		39.8%		39.9%		43.4%	
3	Higher	37.7%		33.0%		41.7%		42.8%		33.2%	
Labor market status^b^											
1	Non-employed	24.6%		26.3%		23.1%		23.2%		25.9%	
2	Self-employed	9.0%		11.6%		6.7%		9.2%		8.7%	
3	Physical work	21.1%		27.6%		15.6%		18.9%		22.8%	
4	Office work	28.0%		16.8%		37.6%		28.4%		27.6%	
5	Knowledge work	17.3%		17.8%		16.9%		20.4%		15.1%	
Equivalent income tertile											
1	Lowest	33.2%		31.9%		34.2%		31.6%		34.7%	
2	Medium	34.9%		33.7%		36.0%		34.8%		35.2%	
3	Highest	32.9%		34.4%		29.7%		33.6%		30.1%	
Economic satisfaction (Satisfied = 1)		82.6%		81.7%		83.4%		84.8%		81.2%	
Gender (Male = 0, reference)		46.0%		100%		0%		47.6%		45.6%	
Age		44.9	25	45.3	25	44.5	25	44.9	25	45.0	25
			64		64		64		64		64
			11.7		11.6		11.7		11.7		11.7
Mental disorder (Yes = 1)		9.8%		10.2%		9.3%		9.0%		11.6%	

^a^ ADHD-PGI = Attention-Deficit/Hyperactivity Disorder polygenic index [11]

^b^ Labor market status encompasses both employment status (designated as category 1 “Non-employed”) and occupational categories (categories 2–5). Category 2, “Self-employed,” includes individuals classified as “Self-employed persons”; category 3, “Physical work,” comprises those classified as “Manual workers”; category 4, “Office work,” includes individuals classified as “Lower-level employees with administrative and clerical occupations”; and category 5, “Knowledge work,” encompasses those classified as “Upper-level employees with administrative, managerial, professional, and related occupations” according to the Statistics Finland registry data

### Statistical analysis

Ordered probit models were used for ordinal outcomes (educational attainment and income tertiles). Binary outcomes—including employment (employed vs. non-employed) and each occupational category (modelled as separate binary indicators)—were analysed using probit models. The models included gender and ADHD-PGI interaction terms to examine potential gender differences in the associations.

For occupational status, we estimated a series of category-specific probit models indicating the probability of selection into each mutually exclusive category. This approach yields marginal effects directly interpretable as percentage-point differences in category-specific probabilities. We note that multinomial logit models can also provide comparable average marginal effects in absolute probability terms and offer a framework for jointly modelling mutually exclusive outcomes. Our specification facilitates a transparent presentation of unconditional associations for each occupational category and does not rely on the independence of irrelevant alternatives (IIA) assumption. However, it does not model the joint probability structure of the outcomes: category probabilities are estimated independently, are not constrained to sum to one, and do not capture substitution patterns across occupational categories. The results should therefore be interpreted as category-specific associations rather than as a complete model of occupational choice.

To evaluate whether ADHD-PGI associations with labour market outcomes extended beyond educational attainment, additional analyses were conducted within the higher education subgroup. This strategy isolates associations conditional on having attained tertiary education, thereby examining whether ADHD genetic liability relates to labour market outcomes beyond educational sorting. Similarly, income was studied within occupational categories to test whether ADHD-PGI was linked to earnings beyond occupational sorting. We also examined economic satisfaction within income categories to assess whether ADHD-PGI was associated with perceived economic well-being among individuals at comparable income levels.

Given the high comorbidity and genetic overlap between ADHD and other psychiatric disorders, a sensitivity analysis was conducted by restricting the sample to individuals without any recorded mental disorder diagnosis. Mental disorder prevalence was identified using lifetime inpatient medical treatment records from the Finnish Care Register for Health Care (HILMO). The binary Mental disorder variable, applied as an exclusion criterion, indicated whether an individual with any Mental, Behavioural, or Neurodevelopmental disorder (F-category diagnoses according to the 10th revision of the International Classification of Diseases [ICD-10], or corresponding diagnoses from earlier ICD editions) had received clinical treatment prior to the study year.

ADHD-PGI was standardised (mean = 0, SD = 1) and primarily analysed in quintiles to allow flexible, distribution-free estimation of associations and to facilitate interpretation of marginal effects across risk strata. Average marginal effects were expressed as percentage-point differences in the probability of each outcome for individuals in ADHD-PGI quintiles 2–5 relative to the lowest quintile (Q1, reference group). To assess robustness and potential non-linearity, we additionally estimated models specifying ADHD-PGI as a continuous linear predictor and as a quadratic term (linear + squared). Results from these alternative specifications are presented in Supplementary Tables 2, 4–5, 7–9, and 11–12. Furthermore, Supplementary Figs. 2–4 display predicted probabilities from probit and ordered probit models using a linear specification of ADHD-PGI. Across outcomes, linear and quadratic models yielded substantively similar results to the quintile analyses, with no consistent evidence of non-linear associations.

Robust standard errors were used to account for potential heteroscedasticity, outliers, non-normality, and within-cluster correlation. Statistical significance was defined as *P* < 0.005, with *P* < 0.05 interpreted as suggestive evidence. Because each model addressed a distinct hypothesis without repeated subgroup comparisons, no additional adjustment for multiple testing was applied. All analyses were conducted using Stata 18 (StataCorp LLC, 2023), following methodological approach consistent with previous studies from our research group examining genetic proxies for psychiatric and brain-related traits in relation to socio-economic outcomes [36, 41–44].

## Results

### ADHD genetic risk and educational attainment

**Fig. 1:**
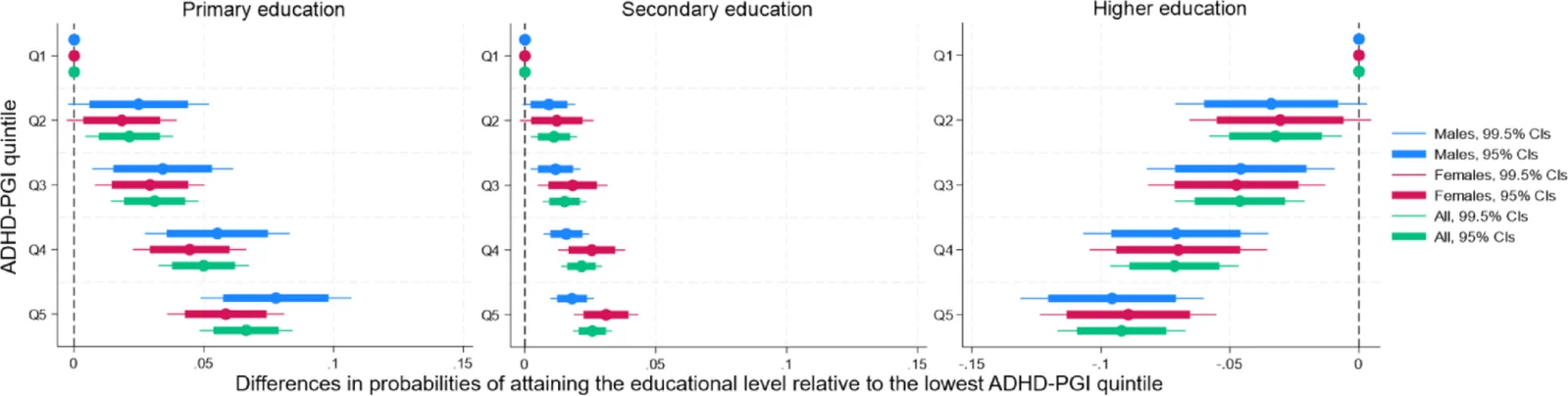
Differences in probabilities of attaining each educational level relative to the lowest ADHD-PGI quintile (*N* = 20,121), with 99.5% and 95% confidence intervals. Note: Average marginal effects from ordered probit models of educational attainment (see Supplemental Table 3). ADHD-PGI = Attention-Deficit/Hyperactivity Disorder polygenic index [11]

ADHD-PGI showed a strong negative association with educational attainment in the full sample, across both genders, and among individuals without diagnosed mental disorders (*P* < 0.001; Supplemental Table 2; Supplemental Fig. 2). No evidence of non-linearity was observed. Individuals in the highest ADHD-PGI quintile were 9.2% points (pp) less likely to attain higher education compared to those in the lowest quintile (*P* < 0.001; 99.5% CI: 6.7–11.7 pp; Fig. 1; Supplemental Table 3).

### ADHD genetic risk and employment

**Fig. 2:**
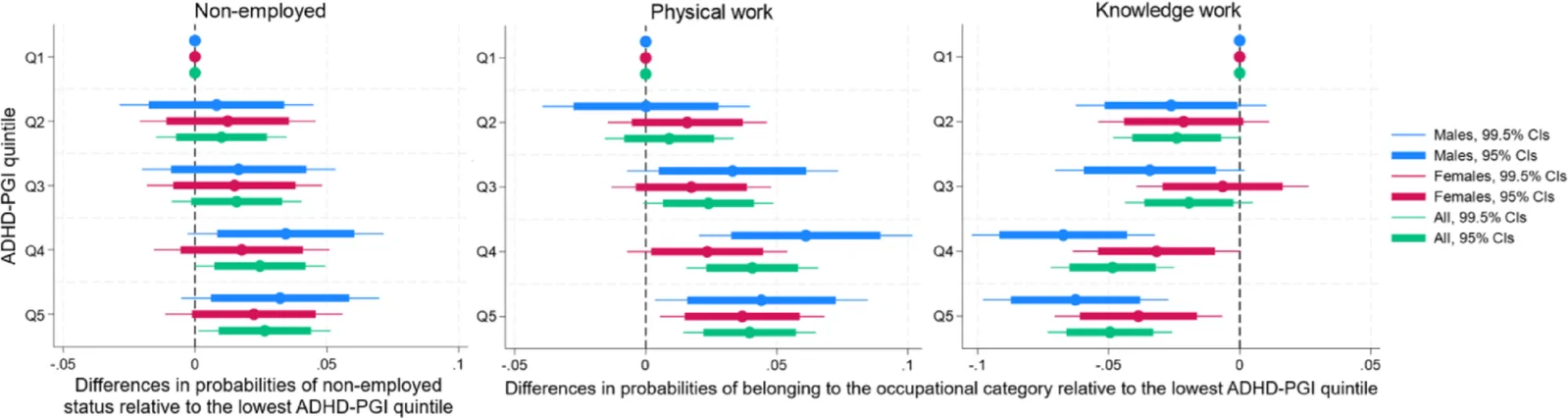
Differences in probabilities of belonging to labour market status categories relative to the lowest ADHD-PGI quintile (*N* = 19,707), with 99.5% and 95% confidence intervals. Note: Average marginal effects from probit models of labour market status (see Supplemental Table 6). ADHD-PGI = Attention-Deficit/Hyperactivity Disorder polygenic index [11]

ADHD-PGI was negatively associated with employment probability in the full sample (*P* < 0.001), and particularly in males (*P* < 0.001), with no evidence of non-linearity (Supplemental Tables 4 & 5; Supplemental Fig. 3). Individuals in the top ADHD-PGI quintile were 2.6 pp less likely to be employed compared to those in the lowest quintile (*P* = 0.003; 99.5% CI: 0.1–5.2 pp; leftmost panel in Fig. 2; Supplemental Table 6).

### ADHD genetic risk and occupational selection

Individuals with higher ADHD-PGI were significantly less likely to be engaged in knowledge work (*P* < 0.001) and more likely to work in physical occupations (*P* < 0.001) across the full sample and within both genders (Supplemental Tables 4 & 5; Supplemental Fig. 3). No non-linear patterns were observed in the association. ADHD-PGI was not significantly related to selection into self-employment or office work. Those in the top ADHD-PGI quintile were 5.0 pp less likely to be in the knowledge work occupational category (*P* < 0.001; 99.5% CI: 2.6–7.3 pp) and 4.0 pp more likely to be in physical work (*P* < 0.001; 99.5% CI: 1.4–6.5 pp; Fig. 2; Supplemental Table 6).

To assess whether these patterns extended beyond selection into higher education, we examined occupational sorting within the higher education subgroup. The negative association between ADHD-PGI and knowledge work persisted in this subgroup (*P* = 0.005), particularly among males, where it was mirrored by a higher likelihood of selection into office work (*P* = 0.014) (Supplemental Tables 7 & 8). This suggests that ADHD-PGI is linked to occupational sorting through pathways that extend beyond educational level.

### ADHD genetic risk and equivalent income

**Fig. 3:**
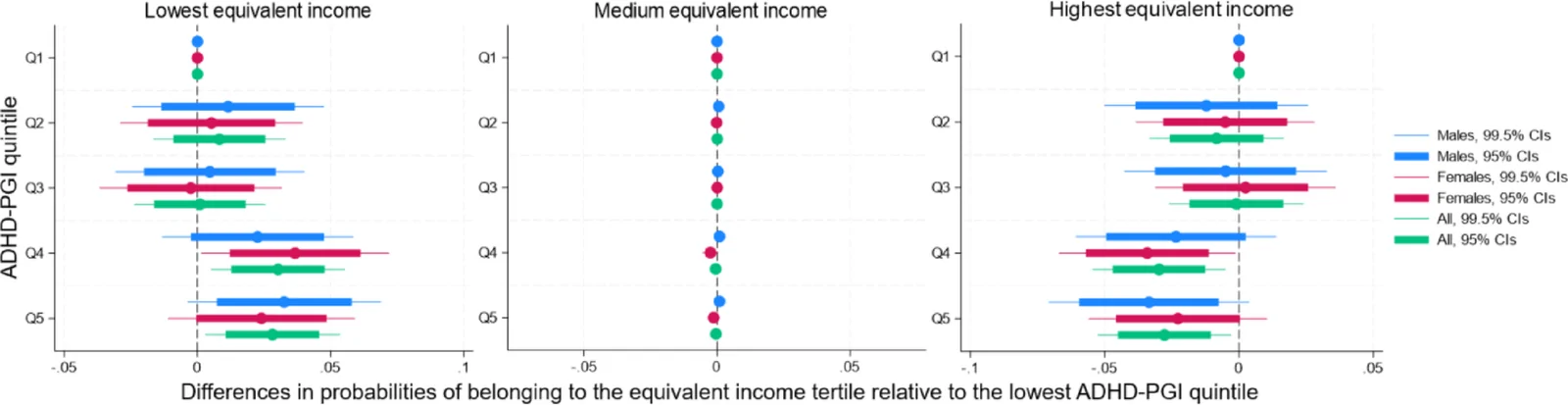
Differences in probabilities of belonging to equivalent income tertiles relative to the lowest ADHD-PGI quintile (*N* = 19,685), with 99.5% and 95% confidence intervals. Note: Average marginal effects from ordered probit models of equivalent income (see Supplemental Table 10). ADHD-PGI = Attention-Deficit/Hyperactivity Disorder polygenic index [11]

Higher ADHD-PGI was associated with lower equivalent income across the full sample and within both genders (*P* < 0.005; Supplemental Table 9; Supplemental Fig. 4), with a more pronounced association observed among males. No non-linear patterns were detected. Individuals in the highest ADHD-PGI quintile were 2.8 pp less likely to be in the top equivalent income tertile compared to those in the lowest quintile (*P* = 0.002; 99.5% CI: 0.3–5.3 pp; Fig. 3; Supplemental Table 10).

Within the higher education subgroup, the negative association between ADHD-PGI and equivalent income remained statistically significant (*P* = 0.006), particularly among males (*P* = 0.005; Supplemental Table 11). However, when examined within specific occupational categories, ADHD-PGI was not significantly associated with income (Supplemental Table 11). This suggests that the observed income disadvantage is primarily driven by educational attainment and occupational sorting rather than earnings penalties within occupational groups.

### ADHD genetic risk and economic satisfaction

**Fig. 4:**
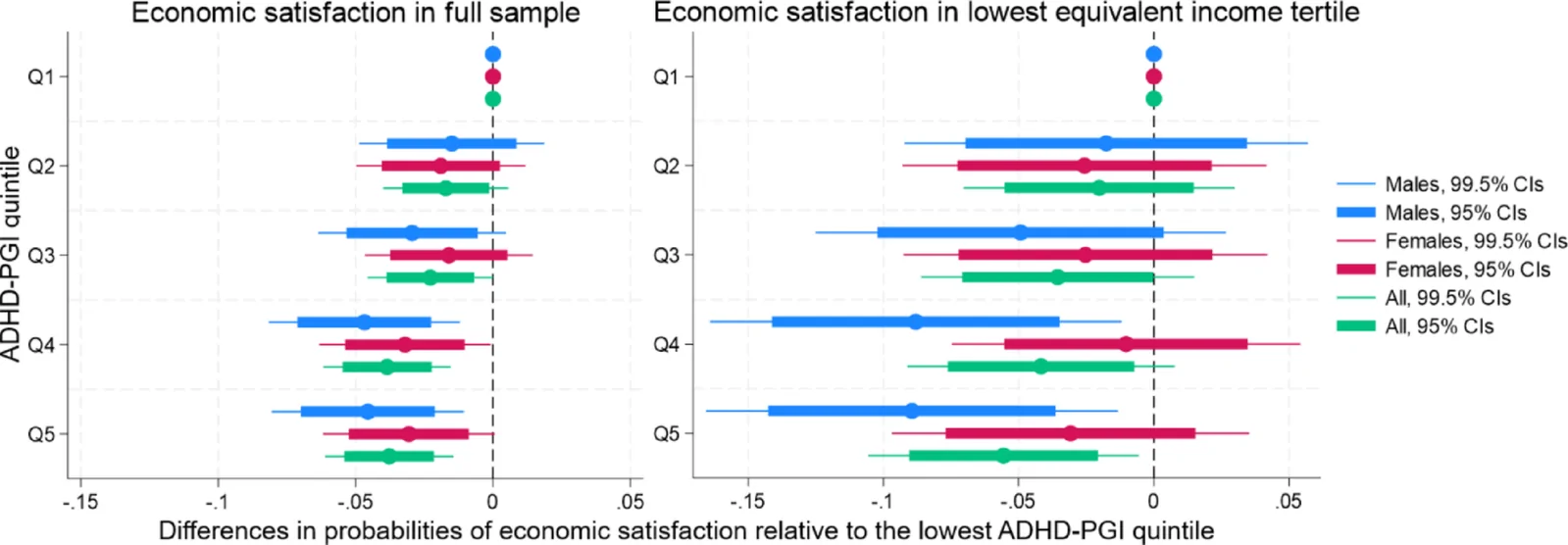
Differences in probabilities of economic satisfaction relative to the lowest ADHD-PGI quintile (*N* = 20,029), with 99.5% and 95% confidence intervals. Note: Average marginal effects from probit models of economic satisfaction (see Supplemental Table 13). ADHD-PGI = Attention-Deficit/Hyperactivity Disorder polygenic index [11]

ADHD-PGI was negatively associated with economic satisfaction across the full sample and within both genders (*P* < 0.001; Supplemental Table 12), with no non-linear effects detected. Those in the highest ADHD-PGI quintile were 3.8 pp less likely to report satisfaction with their economic situation compared to those in the lowest quintile (*P* < 0.001; 99.5% CI: 1.4–6.1 pp; Fig. 4; Supplemental Table 13).

Further analysis showed that this association extended beyond equivalent income tertiles, particularly among males. Within the lowest equivalent income tertile, males with higher ADHD-PGI were significantly less likely to report economic satisfaction (*P* < 0.001; Supplemental Table 12). Specifically, males in the highest ADHD-PGI quintile were 8.9% points less likely to report satisfaction with their economic situation compared to those in the lowest quintile (*P* = 0.001; 99.5% CI: 1.3–16.6 pp; Fig. 4; Supplemental Table 13). In contrast, no corresponding association was observed among females, as suggested by the positive ADHD-PGI × female interaction term and the statistically nonsignificant estimates within the female subsample in the lowest income tertile (Supplemental Table 12).

## Discussion

This study examined how genetic liability for ADHD relates to key sequential economic outcomes—education, employment, occupational selection, income, and economic satisfaction—using data from six Finnish population-based cohorts (1992–2017; *N* = 20,121). The results reveal a consistent pattern of economic disadvantage associated with higher ADHD polygenic risk, suggesting that ADHD-related traits are linked to socio-economic trajectories through multiple, partly independent pathways. While earlier research has documented associations between ADHD genetic liability and individual socioeconomic indicators such as education, income, or employment [16, 18, 19], the present study contributes by examining these outcomes jointly within a sequential framework. This approach allows us to assess how disadvantages associated with ADHD-related traits may accumulate across educational attainment, labour market participation, occupational sorting, and economic well-being.

### ADHD genetic risk and education

Educational attainment is a major determinant of long-term economic success, and we observed a strong negative association between ADHD-PGI and the likelihood of completing higher education. Individuals in the top ADHD-PGI quintile were 9.2 pp less likely to attain higher education than those in the bottom quintile. Given a baseline higher education rate of 37.7% in the sample, this represents a substantial disadvantage. These findings add to prior research showing that ADHD genetic liability is associated with lower academic achievement [16, 18] and a moderate negative genetic correlation between ADHD and educational attainment [12, 14, 15]. They also align with evidence that individuals with ADHD achieve lower educational outcomes [6, 8, 9].

The lack of significant non-linearity indicates that the negative association persists across the genetic risk spectrum. Importantly, similar patterns were observed in the subsample without any lifetime mental disorder diagnosis, supporting the relevance of this association beyond clinical ADHD. Given the strong link between educational attainment and later economic outcomes, this early-life disadvantage likely contributes substantially to downstream inequalities in employment and income in the general population.

### ADHD genetic risk and employment

Higher ADHD-PGI was associated with a greater likelihood of non-employment, particularly among males. Individuals in the highest ADHD-PGI quintile were 2.6 pp less likely to be employed than those in the lowest quintile—a considerable gap given the 24.6% baseline non-employment rate. This is consistent with previous findings that ADHD-PGI is negatively related to employment, largely mediated by education [19], and with observed strong negative genetic correlations between ADHD and socioeconomic status [14]. Individuals with ADHD symptoms also experience higher unemployment rates [4, 6, 9].

Beyond educational differences, ADHD-related characteristics—such as distractibility, impulsivity, and difficulties with sustained attention—may restrict employment opportunities or career stability [7, 8], thereby reinforcing socio-economic disadvantage.

### ADHD genetic risk and occupational selection

ADHD-PGI was strongly associated with occupational sorting. Individuals with higher genetic liability for ADHD were less likely to work in knowledge-based occupations and more likely to be employed in physical labour, with the magnitude of associations differing by gender. Those in the top ADHD-PGI quintile were 5.0 pp less likely to work in knowledge-intensive occupations and 4.0 pp more likely to be in physical work, compared with those in the lowest quintile—substantial differences given baseline participation rates of 17.3% and 21.1%, respectively.

These associations remained within the higher-education subgroup, suggesting that ADHD genetic risk may relate to occupational segregation beyond educational attainment. However, further research using more detailed data on educational quality and field of study is needed to disentangle potential mechanisms.

A range of mechanisms may contribute to the observed associations between ADHD polygenic liability and occupational positioning. One possible interpretation is that individuals with higher ADHD genetic liability may gravitate toward roles with clearer structure or lower cognitive complexity that better align with certain behavioural or attentional profiles [7]. The overrepresentation of high-ADHD-PGI individuals in physical work may reflect not only educational factors but also personal preferences for dynamic, hands-on environments, consistent with the link between ADHD traits and sensation-seeking or preference for immediate rewards [45]. Conversely, knowledge work requires sustained attention and self-regulation—domains often affected by ADHD-related traits [8]—which may be less compatible with such cognitive profiles [46].

At the same time, such patterns may also reflect institutional dynamics within educational systems and labour markets. Neurodivergent individuals may encounter structural barriers during educational progression, including difficulties in highly standardized learning environments, as well as potential stigma, labelling, or discrimination that can influence both educational attainment and subsequent labour market opportunities [7, 47, 48]. These institutional and social processes may interact with individual behavioural traits to shape occupational trajectories. Consequently, the observed occupational sorting likely reflects a combination of person–environment matching, cumulative educational pathways, and structural constraints within labour markets. Recognizing this broader set of mechanisms is important for interpreting the findings and for situating genetic associations within the social and institutional contexts in which educational and labour market outcomes unfold.

### ADHD genetic risk and income

ADHD-PGI was negatively associated with equivalent income. Individuals in the top quintile were 2.8 pp less likely to belong to the highest income tertile—an appreciable gap given the roughly even tertile distribution. This income disadvantage persisted among those with higher education, particularly males, but not within specific occupational categories, suggesting that income disparities primarily reflect differences in educational and occupational sorting rather than within-occupation pay penalties.

These results align with prior findings that ADHD genetic liability and income are inversely related, largely mediated by education [19]. Previous studies report a moderate negative genetic correlation between ADHD and income [13–15] but limited correlation beyond educational attainment [15]. Together with evidence of a causal effect of ADHD liability on reduced household income [16], our findings suggest that ADHD genetic risk contributes to income disparities even in the absence of clinical diagnosis.

The stronger association among males may reflect gendered labour market expectations—where earnings are more directly tied to productivity and employment stability [20]—or gender differences in ADHD symptom expression [24, 25].

### ADHD genetic risk and economic satisfaction

Economic satisfaction represents subjective financial well-being, integrating both objective income and perceived adequacy of resources. Considering subjective alongside objective economic outcomes provides additional insight into the pathways linking ADHD-related traits to socio-economic disadvantage. Objective indicators such as income or employment capture the material conditions available to individuals, whereas subjective evaluations reflect how those conditions are experienced and interpreted.

ADHD-PGI was negatively associated with economic satisfaction, with individuals in the highest quintile 3.8 pp less likely to report being satisfied with their financial situation (baseline 82.6%). This association persisted among males in the lowest income tertile, suggesting that ADHD genetic risk may relate to subjective economic well-being through psychological or behavioural factors beyond objective income.

Traits commonly associated with ADHD—impulsivity, emotional dysregulation, and heightened stress sensitivity [8]—may contribute to reduced satisfaction, independent of financial means. The observed effect among lower-income males could reflect gendered differences in ADHD symptom expression, as men more often exhibit hyperactive–impulsive features [24, 25], which may contribute to financial strain or less effective resource management [49]. Our findings highlight that socio-economic disadvantage associated with ADHD genetic liability may manifest both in measurable economic outcomes and in subjective economic evaluations, which together shape individuals’ overall economic wellbeing.

Taken together, the findings extend the existing literature in several ways. First, by examining multiple economic outcomes within the same population-based sample, the study highlights how associations between ADHD genetic liability and socioeconomic outcomes emerge at different stages of the life course rather than being confined to a single domain. Second, the analysis of occupational categories shows that genetic liability is linked not only to educational attainment but also to occupational positioning, including within the higher-education subgroup. Third, the joint consideration of objective indicators (education, employment, income) and subjective economic satisfaction demonstrates that the socioeconomic implications of ADHD-related traits extend beyond material conditions to individuals’ perceived economic well-being. By integrating these dimensions, the study provides a more comprehensive picture of how ADHD genetic liability may shape economic trajectories in the general population.

### Limitations and future research

This study’s strengths include the use of large, population-based data integrating genetic and socio-economic indicators. Nevertheless, several limitations warrant consideration. First, ADHD-PGI reflects genetic liability for ADHD but shares substantial overlap with other psychiatric and behavioural traits; pleiotropic effects may therefore relate to economic outcomes through shared mechanisms. Second, while polygenic scores mitigate concerns about reverse causality, they do not fully account for gene–environment interactions, which may modify associations. Third, the granularity of available education, occupation, and income data was limited, and future studies with finer data could offer deeper insights. Fourth, as the study sample is Finnish, the findings should be interpreted in light of the specific institutional context of Finland, including its relatively comprehensive welfare state, publicly funded education system, and comparatively regulated labour markets. These institutional features may mitigate some socioeconomic disadvantages associated with neurodevelopmental traits through mechanisms such as universal access to education, income redistribution, and social protection. In settings with weaker social safety nets, higher levels of educational stratification, or more flexible but less protective labour markets, the socioeconomic consequences associated with ADHD-related traits could potentially be more pronounced. Conversely, contexts with stronger inclusion policies or more widespread workplace accommodations for neurodivergent individuals might attenuate some of the observed associations. Future comparative research across countries with differing welfare regimes and labour market institutions would therefore be valuable for assessing the extent to which the patterns observed here reflect general mechanisms linking ADHD-related liability to socioeconomic outcomes versus features specific to the Nordic institutional context.

Future research should explore whether specific educational and workplace environments or practices can mitigate the economic disadvantages associated with ADHD genetic risk. The observed gender differences highlight the need to examine how ADHD liability interacts with gendered labour market trajectories and to identify potential support mechanisms that foster success in knowledge-intensive roles. Examining associations with savings behaviour, financial planning, and long-term wealth accumulation would further clarify the broader economic implications of ADHD genetic liability.

## Conclusion

Genetic liability for ADHD was associated with economic disadvantage across multiple domains, including lower educational attainment, reduced employment, occupational sorting into lower-income jobs, and lower economic satisfaction. The income gap appears to stem primarily from educational and occupational pathways rather than within-occupation earnings differences. Beyond these objective outcomes, higher ADHD genetic risk was linked to less favourable subjective economic wellbeing, highlighting the relevance of considering both dimensions when assessing the broader socio-economic implications of neurodevelopmental traits. Gender differences were evident, with stronger negative effects among males. Recognising these patterns may help inform workplace adaptations, educational support, and policy initiatives to promote more inclusive labour markets that accommodate diverse neurodevelopmental profiles.

## Supplementary Information

Below is the link to the electronic supplementary material.

Supplementary Material 1

## Data Availability

The FR and FH data that support the findings of this study are available from the Finnish Institute for Health and Welfare subject to permission.

## References

[1] Faraone SV, Larsson H (2019) Genetics of attention deficit hyperactivity disorder. Mol Psychiatry 24(4):562–575. 10.1038/s41380-018-0070-0 PMC6477889 29892054

[2] Cortese S, Bellgrove MA, Brikell I, Franke B, Goodman DW, Hartman CA et al (2025) Attention-deficit/hyperactivity disorder (ADHD) in adults: evidence base, uncertainties and controversies. World Psychiatry 24(3):347–371. 10.1002/wps.21374 PMC12434367 40948064

[3] Faraone SV, Bellgrove MA, Brikell I et al (2024) Attention-deficit/hyperactivity disorder. Nat Rev Dis Primers 10(11). 10.1038/s41572-024-00495-0 38388701

[4] Fletcher JM (2014) The effects of childhood ADHD on adult labor market outcomes. Health Econ 23(2):159–181. 10.1002/hec.2907 PMC6714576 23427026

[5] Asherson P, Buitelaar J, Faraone SV, Rohde LA (2016) Adult attention-deficit/hyperactivity disorder: key conceptual issues. Lancet Psychiatry 3(6):568–578. 10.1016/S2215-0366(16)30032-3 27183901

[6] Erskine HE, Norman RE, Ferrari AJ, Chan GCK, Copeland WE, Whiteford HA et al (2016) Long-term outcomes of attention-deficit/hyperactivity disorder and conduct disorder: a systematic review and meta-analysis. J Am Acad Child Adolesc Psychiatry 55(10):841–850. 10.1016/j.jaac.2016.06.016 27663939

[7] Gordon CT, Fabiano GA (2019) The transition of youth with ADHD into the workforce: review and future directions. Clin Child Fam Psychol Rev 22(3):316–347. 10.1007/s10567-019-00274-4 30725305

[8] Faraone SV, Banaschewski T, Coghill D, Zheng Y, Biederman J, Bellgrove MA et al (2021) The World Federation of ADHD international consensus statement: 208 evidence-based conclusions about the disorder. Neurosci Biobehav Rev 128:789–818. 10.1016/j.neubiorev.2021.01.022 PMC8328933 33549739

[9] Jangmo A, Kuja-Halkola R, Pérez-Vigil A, Almqvist C, Bulik CM, D’Onofrio B et al (2021) Attention-deficit/hyperactivity disorder and occupational outcomes: the role of educational attainment, comorbid developmental disorders, and intellectual disability. PLoS ONE 16(3):e0247724. 10.1371/journal.pone.0247724 PMC7968636 33730071

[10] Loewenstein G, Wojtowicz Z (2025) The economics of attention. J Econ Lit 63(3):1038–1089. 10.1257/jel.20241665

[11] Demontis D, Walters RK, Martin J, Mattheisen M, Als TD et al (2019) Discovery of the first genome-wide significant risk loci for ADHD. Nat Genet 51(1):63–75. 10.1038/s41588-018-0269-7 PMC6481311 30478444

[12] Demontis D, Walters GB, Athanasiadis G, Walters R, Therrien K, Nielsen TT et al (2023) Genome-wide analyses of ADHD identify 27 risk loci, refine the genetic architecture and implicate several cognitive domains. Nat Genet 55(2):198–208. 10.1038/s41588-022-01285-8 PMC10914347 36702997

[13] Hill WD, Davies NM, Ritchie SJ et al (2019) Genome-wide analysis identifies molecular systems and 149 genetic loci associated with income. Nat Commun 10:5741. 10.1038/s41467-019-13585-5 PMC6915786 31844048

[14] Marees AT, Smit DJA, Abdellaoui A, Nivard MG, van den Brink W, Denys D et al (2021) Genetic correlates of socio-economic status influence the pattern of shared heritability across mental health traits. Nat Hum Behav 5(8):1065–1073. 10.1038/s41562-021-01053-4 PMC8376746 33686200

[15] Kweon H, Burik CA, Ning Y, Ahlskog R, Xia C, Abner E et al Associations between common genetic variants and income provide insights about the socio-economic health gradient. Nat Hum Behav. 2025;online ahead of print. 10.1038/s41562-024-02080-7 PMC12018258 39875632

[16] Soler Artigas M, Sánchez-Mora C, Rovira P, Vilar-Ribó L, Ramos-Quiroga JA, Ribasés M (2023) Mendelian randomization analysis for attention deficit/hyperactivity disorder: studying a broad range of exposures and outcomes. Int J Epidemiol 52(2):386–402. 10.1093/ije/dyac128 PMC10114062 35690959

[17] Thorell LB, Bohlin G, Rydell AM (2004) Two types of inhibitory control: predictive relations to social functioning. Int J Behav Dev 28(3):193–203. 10.1080/01650250344000389

[18] Stergiakouli E, Martin J, Hamshere ML, Heron J, St Pourcain B, Timpson NJ et al (2017) Association between polygenic risk scores for attention-deficit/hyperactivity disorder and educational and cognitive outcomes in the general population. Int J Epidemiol 46(2):421–428. 10.1093/ije/dyw216 PMC5424076 27694570

[19] Rietveld CA, Patel PC (2019) ADHD and later-life labor market outcomes in the United States. Eur J Health Econ 20(7):949–967. 10.1007/s10198-019-01055-0 PMC6687698 31049764

[20] Rucklidge JJ (2008) Gender differences in ADHD: implications for psychosocial treatments. Expert Rev Neurother 8(4):643–. 10.1586/14737175.8.4.643. 55 18416665

[21] Autor D (2019) Work of the past, work of the future. AEA Pap Proc. ;109:1–32. 10.1257/pandp.20191110

[22] Martin J, Walters RK, Demontis D, Mattheisen M, Lee SH et al (2021) A genetic investigation of sex bias in the prevalence of attention-deficit/hyperactivity disorder. Biol Psychiatry 89(5):450–457. 10.1016/j.biopsych.2017.11.026 PMC5992329 29325848

[23] Ribasés M, Mitjans M, Hartman CA, Soler Artigas M, Demontis D, Larsson H et al (2023) Genetic architecture of ADHD and overlap with other psychiatric disorders and cognition-related phenotypes. Neurosci Biobehav Rev 153:105313. 10.1016/j.neubiorev.2023.105313 PMC10789879 37451654

[24] Williams T, Horstmann L, Kayani L, Lim AXH, Russell A, Ford T et al (2025) An item-level systematic review of the presentation of ADHD in females. Neurosci Biobehav Rev 171:106064. 10.1016/j.neubiorev.2025.106064 39952337

[25] Mestres F, Richarte V, Crespín JJ, Torrent C, Biel S, Ramos C et al (2025) Sex differences in adults with attention-deficit/hyperactivity disorder: a population-based study. Eur Psychiatry 68(1):e90. 10.1192/j.eurpsy.2025.2441 PMC12260720 40211765

[26] Solberg BS, Halmøy A, Engeland A, Igland J, Haavik J, Klungsøyr K (2018) Gender differences in psychiatric comorbidity: a population-based study of 40 000 adults with attention deficit hyperactivity disorder. Acta Psychiatr Scand 137(3):176–186. 10.1111/acps.12845 PMC5838558 29266167

[27] Becker J, Burik CA, Goldman G, Wang N, Jayashankar H, Bennett M et al (2021) Resource profile and user guide of the Polygenic Index Repository. Nat Hum Behav 5(12):1744–1758. 10.1038/s41562-021-01119-3 PMC8678380 34140656

[28] Garcia-Argibay M, Brikell I, Thapar A, Lichtenstein P, Lundström S, Demontis D et al (2024) Attention-deficit/hyperactivity disorder and major depressive disorder: evidence from multiple genetically informed designs. Biol Psychiatry 95(5):444–452. 10.1016/j.biopsych.2023.07.017 37562520

[29] Meisinger C, Freuer D (2023) Understanding the causal relationships of attention-deficit/hyperactivity disorder with mental disorders and suicide attempt: a network Mendelian randomisation study. BMJ Ment Health 26(1):e300642. 10.1136/bmjment-2022-300642 PMC11146378 37669871

[30] Kittel-Schneider S, Arteaga-Henriquez G, Vasquez AA, Asherson P, Banaschewski T, Brikell I et al (2022) Non-mental diseases associated with ADHD across the lifespan: Fidgety Philipp and Pippi Longstocking at risk of multimorbidity? Neurosci Biobehav Rev 132:1157–1180. 10.1016/j.neubiorev.2021.10.035 34757108

[31] Kessler RC, Adler LA, Barkley R, Biederman J, Conners CK, Faraone SV et al (2005) Patterns and predictors of Attention-Deficit/Hyperactivity Disorder persistence into adulthood: results from the National Comorbidity Survey Replication. Biol Psychiatry 57(11):1442–1451. 10.1016/j.biopsych.2005.04.001 PMC2847347 15950019

[32] Sobanski E (2006) Psychiatric comorbidity in adults with attention-deficit/hyperactivity disorder (ADHD). Eur Arch Psychiatry Clin Neurosci 256(Suppl 1):i26–31. 10.1007/s00406-006-1004-4 16977548

[33] Kessler RC, Adler LA, Berglund P, Green JG, McLaughlin KA, Fayyad J et al (2014) The effects of temporally secondary co-morbid mental disorders on the associations of DSM-IV ADHD with adverse outcomes in the US NCS-A Adolescent Supplement. Psychol Med 44(8):1779–1792. 10.1017/S0033291713002419 PMC4124915 24103255

[34] Borodulin K, Tolonen H, Jousilahti P, Jula A, Juolevi A, Koskinen S et al (2018) Cohort profile: the national FINRISK study. Int J Epidemiol 47(3):696–i. 10.1093/ije/dyx239 29165699

[35] Borodulin K, Sääksjärvi K (2017) FinHealth 2017 study: methods. Finnish Institute for Health and Welfare, Helsinki

[36] Hazak A, Liuhanen J, Kantojärvi K, Sulkava S, Jääskeläinen T, Salomaa V et al (2025) Schizophrenia genetic risk and labour market outcomes in the Finnish general population: are schizophrenia-related traits penalised or rewarded? Compr Psychiatry 152600. 10.1016/j.comppsych.2025.152600 40319553

[37] Paukkeri T, Ravaska T, Riihelä M (2024) Examining inequalities: from labour markets to social outcomes in Finland. Fisc Stud 45(2):225–241. 10.1111/1475-5890.12369

[38] Reinikainen J, Tolonen H, Borodulin K, Härkänen T, Jousilahti P, Karvanen J et al (2018) Participation rates by educational levels have diverged during 25 years in Finnish health examination surveys. Eur J Public Health 28(2):237–243. 10.1093/eurpub/ckx151 29036286

[39] Kyyrä T, Pesola H (2020) The labor market in Finland, 2000–2018. IZA World of Labor

[40] OECD (2013) OECD framework for statistics on the distribution of household income, consumption and wealth. Chapter 8: framework for integrated analysis. OECD Publishing, Paris

[41] Hazak A, Kantojärvi K, Sulkava S, Kukk M, Jääskeläinen T, Salomaa V et al (2025) Genetic disparities in sleep traits and human capital development: a 25-year study in Finnish population-based cohorts. Scand J Work Environ Health 52(1):63. 10.5271/sjweh.4255 PMC12782571 41084904

[42] Hazak A, Kantojärvi K, Liuhanen J, Sulkava S, Jääskeläinen T, Salomaa V et al (2025) Genetic predisposition for morningness-eveningness and economic disadvantage: Evidence from Finland over 25 years. Sleep Med 106811. 10.1016/j.sleep.2025.106811 40972070

[43] Hazak A, Liuhanen J, Kantojärvi K, Kukk M, Sulkava S, Jääskeläinen T et al (2026) Polygenic Risks for Mood Disorders and Economic Well-being: Study of Finnish Cohorts. Depress Anxiety 1008569. 10.1155/da/1008569 PMC12927894 41737354

[44] Hazak A, Liuhanen J, Kantojärvi K, Sulkava S, Jääskeläinen T, Salomaa V, Koskinen S, Perola M, Paunio T (2026) Neuroticism genetic risk, gender, and economic disadvantage. Eur J Pers. 10.1177/08902070261473064

[45] Dalley JW, Robbins TW (2017) Fractionating impulsivity: neuropsychiatric implications. Nat Rev Neurosci 18(3):158–171. 10.1038/nrn.2017.8 28209979

[46] Heckman JJ, Kautz T (2012) Hard evidence on soft skills. Labour Econ 19:451–464. 10.1016/j.labeco.2012.05.014 PMC3612993 23559694

[47] Bisset M, Winter L, Middeldorp CM, Coghill D, Zendarski N, Bellgrove MA, Sciberras E (2022) Recent Attitudes toward ADHD in the Broader Community: A Systematic Review. J Atten Disord 26(4):537–548. 10.1177/10870547211003671 33769111

[48] Vargas-Salas O, Alcazar-Gonzales JC, Fernández-Fernández FA, Molina-Rodríguez FN, Paredes-Velazco R, Carcausto-Zea ML (2025) Neurodivergence and the Workplace: A Systematic Review of the Literature. J Vocat Rehabil 63(1):83–94. 10.1177/10522263251337564

[49] Bangma DF, Koerts J, Fuermaier ABM, Mette C, Zimmermann M, Toussaint AK et al (2019) Financial decision-making in adults with ADHD. Neuropsychology 33(8):1065–1076. 10.1037/neu0000571 31343233

