# Supplementary material for: Associations of ADHD genetic risk with objective and subjective economic wellbeing: evidence from Finnish population-based cohorts
Source: Soc Psychiatry Psychiatr Epidemiol. 2026 Sep 1;61(11):2461–73. doi: 10.1007/s00127-026-03183-4 (PMC13694503; doi:10.1007/s00127-026-03183-4)
Supplement: Supplementary file 1 [file 127_2026_3183_MOESM1_ESM.docx]

**Supplemental Material**

**Supplemental Fig. 1.** Histogram of ADHD-PGI by gender

*Note: ADHD-PGI = Attention-Deficit/Hyperactivity Disorder polygenic index [11]; Q1–Q5 = ADHD-PGI quintiles.*

**Supplemental Fig. 2.** ADHD-PGI and predicted probabilities of attaining a specific educational level with 99.5% CI

*Note: ADHD-PGI = Attention-Deficit/Hyperactivity Disorder polygenic index [11]; Figure based on predicted probabilities from ordered probit regression models of educational level, as detailed in Supplemental Table 2, using a linear specification of the polygenic score as the main explanatory variable.*

**Supplemental Fig. 3.** ADHD-PGI and predicted probabilities of labour market status with 99.5% CI

*Note: ADHD-PGI = Attention-Deficit/Hyperactivity Disorder polygenic index [11]; Figures based on predicted probabilities from probit regression models of labour market status, as detailed in Supplemental Table 5, using a linear specification of the polygenic score as the main explanatory variable.*

**Supplemental Fig. 4.** ADHD-PGI and predicted probabilities of equivalent income tertile with 99.5% CI

*Note: ADHD-PGI = Attention-Deficit/Hyperactivity Disorder polygenic index [11]; Figure based on predicted probabilities from ordered probit regression models of equivalent income tertile, as detailed in Supplemental Table 9, using a linear specification of the polygenic score as the main explanatory variable.*

**Supplemental Table 1.** Pairwise correlation coefficients and their statistical significance

|  | ADHD-PGI | Educational level | Labor market status:  Non-employed | Labor market status:  Self-employed | Labor market status:  Physical work | Labor market status:  Office work | Labor market status:  Knowledge work | Equivalent income tertiles | Economic satisfaction | Gender | Age | Birth cohort | Year | PC1 | PC2 |
| --- | --- | --- | --- | --- | --- | --- | --- | --- | --- | --- | --- | --- | --- | --- | --- |
|  |  |  |  |  |  |  |  |  |  |  |  |  |  |  |  |
| Educational level | -0.076 |  |  |  |  |  |  |  |  |  |  |  |  |  |  |
|  | 0.000 |  |  |  |  |  |  |  |  |  |  |  |  |  |  |
|  |  |  |  |  |  |  |  |  |  |  |  |  |  |  |  |
| Labor market status: | 0.025 | -0.206 |  |  |  |  |  |  |  |  |  |  |  |  |  |
| Non-employed | 0.000 | 0.000 |  |  |  |  |  |  |  |  |  |  |  |  |  |
|  |  |  |  |  |  |  |  |  |  |  |  |  |  |  |  |
| Labor market status: | -0.007 | -0.059 | -0.175 |  |  |  |  |  |  |  |  |  |  |  |  |
| Self-employed | 0.292 | 0.000 | 0.000 |  |  |  |  |  |  |  |  |  |  |  |  |
|  |  |  |  |  |  |  |  |  |  |  |  |  |  |  |  |
| Labor market status: | 0.033 | -0.266 | -0.288 | -0.158 |  |  |  |  |  |  |  |  |  |  |  |
| Physical work | 0.000 | 0.000 | 0.000 | 0.000 |  |  |  |  |  |  |  |  |  |  |  |
|  |  |  |  |  |  |  |  |  |  |  |  |  |  |  |  |
| Labor market status: | -0.007 | 0.143 | -0.346 | -0.191 | -0.314 |  |  |  |  |  |  |  |  |  |  |
| Office work | 0.293 | 0.000 | 0.000 | 0.000 | 0.000 |  |  |  |  |  |  |  |  |  |  |
|  |  |  |  |  |  |  |  |  |  |  |  |  |  |  |  |
| Labor market status: | -0.051 | 0.400 | -0.255 | -0.140 | -0.231 | -0.278 |  |  |  |  |  |  |  |  |  |
| Knowledge work | 0.000 | 0.000 | 0.000 | 0.000 | 0.000 | 0.000 |  |  |  |  |  |  |  |  |  |
|  |  |  |  |  |  |  |  |  |  |  |  |  |  |  |  |
| Equivalent income | -0.032 | 0.293 | -0.361 | -0.026 | -0.035 | 0.141 | 0.327 |  |  |  |  |  |  |  |  |
| tertiles | 0.000 | 0.000 | 0.000 | 0.000 | 0.000 | 0.000 | 0.000 |  |  |  |  |  |  |  |  |
|  |  |  |  |  |  |  |  |  |  |  |  |  |  |  |  |
| Economic satisfaction | -0.032 | 0.050 | -0.159 | 0.020 | 0.016 | 0.054 | 0.103 | 0.270 |  |  |  |  |  |  |  |
|  | 0.000 | 0.000 | 0.000 | 0.004 | 0.026 | 0.000 | 0.000 | 0.000 |  |  |  |  |  |  |  |
|  |  |  |  |  |  |  |  |  |  |  |  |  |  |  |  |
| Gender | 0.011 | 0.083 | -0.036 | -0.085 | -0.145 | 0.227 | -0.011 | -0.043 | 0.021 |  |  |  |  |  |  |
| (Male = 0, Female = 1) | 0.114 | 0.000 | 0.000 | 0.000 | 0.000 | 0.000 | 0.106 | 0.000 | 0.003 |  |  |  |  |  |  |
|  |  |  |  |  |  |  |  |  |  |  |  |  |  |  |  |
| Age | 0.005 | -0.263 | 0.212 | 0.031 | -0.071 | -0.097 | -0.055 | 0.044 | 0.098 | -0.035 |  |  |  |  |  |
|  | 0.460 | 0.000 | 0.000 | 0.000 | 0.000 | 0.000 | 0.000 | 0.000 | 0.000 | 0.000 |  |  |  |  |  |
|  |  |  |  |  |  |  |  |  |  |  |  |  |  |  |  |
| Birth cohort | -0.003 | 0.341 | -0.199 | -0.021 | 0.032 | 0.085 | 0.092 | -0.029 | -0.064 | 0.023 | -0.795 |  |  |  |  |
| (dummies, 1920ies to 2000s) | 0.640 | 0.000 | 0.000 | 0.003 | 0.000 | 0.000 | 0.000 | 0.000 | 0.000 | 0.001 | 0.000 |  |  |  |  |
|  |  |  |  |  |  |  |  |  |  |  |  |  |  |  |  |
| Year | 0.006 | 0.221 | -0.052 | 0.007 | -0.046 | 0.014 | 0.081 | 0.021 | 0.028 | -0.011 | 0.038 | 0.540 |  |  |  |
| (study year, 1992 to 2017) | 0.438 | 0.000 | 0.000 | 0.326 | 0.000 | 0.044 | 0.000 | 0.004 | 0.000 | 0.122 | 0.000 | 0.000 |  |  |  |
|  |  |  |  |  |  |  |  |  |  |  |  |  |  |  |  |
| PC1 | -0.042 | 0.056 | -0.061 | -0.014 | -0.015 | 0.009 | 0.079 | 0.080 | -0.015 | -0.002 | -0.008 | 0.032 | 0.046 |  |  |
|  | 0.000 | 0.000 | 0.000 | 0.055 | 0.030 | 0.192 | 0.000 | 0.000 | 0.038 | 0.835 | 0.276 | 0.000 | 0.000 |  |  |
|  |  |  |  |  |  |  |  |  |  |  |  |  |  |  |  |
| PC2 | 0.026 | -0.045 | 0.000 | -0.004 | 0.013 | -0.001 | -0.009 | -0.014 | -0.017 | 0.005 | 0.007 | -0.063 | -0.102 | 0.044 |  |
|  | 0.000 | 0.000 | 0.958 | 0.531 | 0.072 | 0.931 | 0.186 | 0.049 | 0.015 | 0.446 | 0.346 | 0.000 | 0.000 | 0.000 |  |
|  |  |  |  |  |  |  |  |  |  |  |  |  |  |  |  |
| PC3 | 0.036 | 0.010 | 0.009 | -0.010 | -0.004 | 0.007 | -0.008 | -0.029 | 0.012 | 0.010 | -0.027 | 0.039 | 0.033 | 0.001 | 0.035 |
|  | 0.000 | 0.173 | 0.199 | 0.177 | 0.610 | 0.325 | 0.233 | 0.000 | 0.090 | 0.171 | 0.000 | 0.000 | 0.000 | 0.935 | 0.000 |

*Note: ADHD-PGI = Attention-Deficit/Hyperactivity Disorder polygenic index [11]*

**Supplemental Table 2.** Coefficient estimates from ordered probit models of education

| **Sample** | **All** | **Males** | **Females** | **All** | **All, no mental disorder** |
| --- | --- | --- | --- | --- | --- |
| **ADHD-PGI specification** | **Linear** | **Linear** | **Linear** | **Linear + squared** | **Linear** |
| N | 20,121 | 9,248 | 10,873 | 20,121 | 18,158 |
| Model | oprobit | oprobit | oprobit | oprobit | oprobit |
| **Dependent variable: Education** |  |  |  |  |  |
| Explanatory variables: |  |  |  |  |  |
| ADHD-PGI | -0.101*** | -0.100*** | -0.092*** | -0.095*** | -0.097*** |
|  | (0.012) | (0.012) | (0.011) | (0.008) | (0.013) |
| ADHD-PGI squared |  |  |  | -0.004 |  |
|  |  |  |  | (0.006) |  |
| Female | 0.202*** |  |  | 0.202*** | 0.190*** |
|  | (0.016) |  |  | (0.016) | (0.017) |
| ADHD-PGI # Female | 0.012 |  |  |  | 0.005 |
|  | (0.016) |  |  |  | (0.017) |
| Age | 0.008 | 0.006 | 0.013 | 0.008 | 0.009 |
|  | (0.007) | (0.010) | (0.010) | (0.007) | (0.008) |
| Age squared | -0.000*** | -0.000* | -0.001*** | -0.000*** | -0.000*** |
|  | (0.000) | (0.000) | (0.000) | (0.000) | (0.000) |
| PC1 | 8.482*** | 11.078*** | 5.998*** | 8.492*** | 8.637*** |
|  | (1.157) | (1.670) | (1.611) | (1.157) | (1.220) |
| PC2 | -3.330* | -1.869 | -4.904** | -3.321* | -3.581** |
|  | (1.197) | (1.661) | (1.712) | (1.197) | (1.248) |
| PC3 | 0.131 | -0.098 | 0.498 | 0.125 | 0.893 |
|  | (1.112) | (1.585) | (1.566) | (1.112) | (1.179) |
| Birth cohort dummies | Yes | Yes | Yes | Yes | Yes |
| Year dummies | Yes | Yes | Yes | Yes | Yes |
|  |  |  |  |  |  |
| Cut: Primary/Secondary education | -1.030*** | -0.988*** | -1.191*** | -1.033*** | -1.077*** |
|  | (0.174) | (0.250) | (0.244) | (0.174) | (0.184) |
| Cut: Secondary/Higher education | 0.252 | 0.307 | 0.089 | 0.249 | 0.204 |
|  | (0.175) | (0.251) | (0.244) | (0.175) | (0.184) |
|  |  |  |  |  |  |
| Pseudo-R^2^ | 0.078 | 0.051 | 0.100 | 0.078 | 0.080 |
| p(χ^2^) | *** | *** | *** | *** | *** |

** P < 0.05, ** P < 0.005, *** P < 0.001; robust SE in parentheses; ADHD-PGI = Attention-Deficit/Hyperactivity Disorder polygenic index [11]; the binary Mental disorder variable, used as an exclusion criterion for the results presented in the last column, indicates whether the individual had a recorded diagnosis of any Mental, Behavioural, or Neurodevelopmental disorder (F-category diagnoses under the 10th revision of the International Classification of Diseases [ICD-10] or corresponding diagnoses from earlier ICD editions) prior to the survey year in the Finnish Care Register for Health Care (HILMO).*

**Supplemental Table 3.** Differences in predicted probabilities of attaining each educational level across ADHD-PGI quintiles relative to the lowest quintile

| **Sample** | **All** | **Males** | **Females** |
| --- | --- | --- | --- |
| N | 20,121 | 9,248 | 10,873 |
| Average marginal effects: |  |  |  |
| **ADHD-PGI Q1** | Reference | Reference | Reference |
| **ADHD-PGI Q2** |  |  |  |
| Primary education | 0.021*** | 0.025* | 0.018* |
|  | (0.006) | (0.010) | (0.008) |
| Secondary education | 0.011*** | 0.009* | 0.012* |
|  | (0.003) | (0.004) | (0.005) |
| Higher education | -0.032*** | -0.034* | -0.031* |
|  | (0.009) | (0.013) | (0.013) |
| **ADHD-PGI Q3** |  |  |  |
| Primary education | 0.031*** | 0.034*** | 0.029*** |
|  | (0.006) | (0.010) | (0.008) |
| Secondary education | 0.015*** | 0.012*** | 0.018*** |
|  | (0.003) | (0.003) | (0.005) |
| Higher education | -0.046*** | -0.046*** | -0.047*** |
|  | (0.009) | (0.013) | (0.012) |
| **ADHD-PGI Q4** |  |  |  |
| Primary education | 0.050*** | 0.055*** | 0.045*** |
|  | (0.006) | (0.010) | (0.008) |
| Secondary education | 0.022*** | 0.016*** | 0.026*** |
|  | (0.003) | (0.003) | (0.005) |
| Higher education | -0.072*** | -0.071*** | -0.070*** |
|  | (0.009) | (0.013) | (0.012) |
|  |  |  |  |
| **ADHD-PGI Q5** |  |  |  |
| Primary education | 0.066*** | 0.078*** | 0.058*** |
|  | (0.006) | (0.010) | (0.008) |
| Secondary education | 0.026*** | 0.018*** | 0.031*** |
|  | (0.003) | (0.003) | (0.004) |
| Higher education | -0.092*** | -0.096*** | -0.089*** |
|  | (0.009) | (0.013) | (0.012) |

** P < 0.05, ** P < 0.005, *** P < 0.001; robust SE in parentheses; ADHD-PGI = Attention-Deficit/Hyperactivity Disorder polygenic index [11]. Average marginal effects from ordered probit models of educational attainment, with ADHD-PGI quintiles as the main explanatory variable. Models control for gender, age (linear and squared), birth cohort dummies, study year dummies, and the first three genetic principal components (PC1–PC3).*

**Supplemental Table 4.** Coefficient estimates from probit models of labour market status in the full sample

| **Sample** | **All** | **All** | **All** | **All** | **All** | **All** | **All** | **All** | **All** | **All** |
| --- | --- | --- | --- | --- | --- | --- | --- | --- | --- | --- |
| **Labour market status** | **Non-employed** | **Self-employed** | **Physical**  **work** | **Office**  **work** | **Knowledge work** | **Non-employed** | **Self-employed** | **Physical**  **work** | **Office**  **work** | **Knowledge work** |
| **ADHD-PGI specification** | **Linear** | **Linear** | **Linear** | **Linear** | **Linear** | **Linear + squared** | **Linear + squared** | **Linear + squared** | **Linear + squared** | **Linear + squared** |
| N | 19,707 | 19,707 | 19,707 | 19,707 | 19,707 | 19,707 | 19,707 | 19,707 | 19,707 | 19,707 |
| Model | probit | probit | probit | probit | probit | probit | probit | probit | probit | probit |
| **Dependent variable: belonging to the specific labour market status** | | | | | | | | | | |
| Explanatory variables: | | | | | | | | | | |
| ADHD-PGI | 0.053*** | -0.020 | 0.053*** | -0.015 | -0.095*** | 0.040*** | -0.012 | 0.051*** | -0.013 | -0.076*** |
|  | (0.015) | (0.017) | (0.014) | (0.016) | (0.016) | (0.010) | (0.013) | (0.010) | (0.010) | (0.011) |
| ADHD-PGI squared |  |  |  |  |  | 0.003 | 0.002 | -0.005 | -0.001 | -0.002 |
|  |  |  |  |  |  | (0.007) | (0.009) | (0.007) | (0.007) | (0.008) |
| Female | -0.106*** | -0.296*** | -0.426*** | 0.657*** | -0.032 | -0.106*** | -0.296*** | -0.426*** | 0.657*** | -0.034 |
|  | (0.021) | (0.026) | (0.021) | (0.020) | (0.022) | (0.021) | (0.026) | (0.021) | (0.020) | (0.022) |
| ADHD-PGI # Female | -0.025 | 0.019 | -0.004 | 0.003 | 0.038 |  |  |  |  |  |
|  | (0.021) | (0.026) | (0.021) | (0.020) | (0.022) |  |  |  |  |  |
| Age | -0.256*** | 0.123*** | 0.049*** | 0.066*** | 0.127*** | -0.256*** | 0.123*** | 0.049*** | 0.066*** | 0.127*** |
|  | (0.010) | (0.013) | (0.010) | (0.009) | (0.010) | (0.010) | (0.013) | (0.010) | (0.009) | (0.010) |
| Age squared | 0.003*** | -0.001*** | -0.001*** | -0.001*** | -0.002*** | 0.003*** | -0.001*** | -0.001*** | -0.001*** | -0.002*** |
|  | (0.000) | (0.000) | (0.000) | (0.000) | (0.000) | (0.000) | (0.000) | (0.000) | (0.000) | (0.000) |
| PC1 | -12.655*** | -3.276 | -2.222 | 2.264 | 16.097*** | -12.659*** | -3.281 | -2.213 | 2.267 | 16.096*** |
|  | (1.505) | (1.809) | (1.435) | (1.363) | (1.475) | (1.504) | (1.810) | (1.436) | (1.363) | (1.475) |
| PC2 | 0.603 | -0.685 | 1.453 | -0.604 | -1.430 | 0.611 | -0.703 | 1.479 | -0.601 | -1.420 |
|  | (1.692) | (1.872) | (1.469) | (1.374) | (1.445) | (1.693) | (1.870) | (1.469) | (1.374) | (1.445) |
| PC3 | 3.778* | -2.080 | -0.677 | 0.256 | -2.247 | 3.792* | -2.103 | -0.668 | 0.255 | -2.281 |
|  | (1.479) | (1.808) | (1.454) | (1.317) | (1.389) | (1.480) | (1.807) | (1.454) | (1.317) | (1.389) |
| Birth cohort dummies | Yes | Yes | Yes | Yes | Yes | Yes | Yes | Yes | Yes | Yes |
| Year dummies | Yes | Yes | Yes | Yes | Yes | Yes | Yes | Yes | Yes | Yes |
|  |  |  |  |  |  |  |  |  |  |  |
| Constant | 3.694*** | -3.902*** | -1.179*** | -1.761*** | -3.542*** | 3.693*** | -3.905*** | -1.174*** | -1.760*** | -3.541*** |
|  | (0.240) | (0.312) | (0.238) | (0.226) | (0.250) | (0.240) | (0.312) | (0.238) | (0.227) | (0.250) |
|  |  |  |  |  |  |  |  |  |  |  |
| Pseudo-R^2^ | 0.129 | 0.027 | 0.035 | 0.067 | 0.038 | 0.129 | 0.027 | 0.035 | 0.067 | 0.037 |
| p(χ^2^) | *** | *** | *** | *** | *** | *** | *** | *** | *** | *** |

** P < 0.05, ** P < 0.005, *** P < 0.001; robust SE in parentheses; ADHD-PGI = Attention-Deficit/Hyperactivity Disorder polygenic index [11].*

**Supplemental Table 5.** Coefficient estimates from probit models of labour market status by gender

| **Sample** | **Males** | **Males** | **Males** | **Males** | **Males** | **Females** | **Females** | **Females** | **Females** | **Females** |
| --- | --- | --- | --- | --- | --- | --- | --- | --- | --- | --- |
| **Labour market status** | **Non-employed** | **Self-employed** | **Physical**  **work** | **Office**  **work** | **Knowledge work** | **Non-employed** | **Self-employed** | **Physical**  **work** | **Office**  **work** | **Knowledge work** |
| **ADHD-PGI specification** | **Linear** | **Linear** | **Linear** | **Linear** | **Linear** | **Linear** | **Linear** | **Linear** | **Linear** | **Linear** |
| N | 9,068 | 9,068 | 9,068 | 9,068 | 9,068 | 10,639 | 10,639 | 10,639 | 10,639 | 10,639 |
| Model | probit | probit | probit | probit | probit | probit | probit | probit | probit | probit |
| **Dependent variable: belonging to the specific labour market status** | | | | | | | | | | |
| Explanatory variables: | | | | | | | | | | |
| ADHD-PGI | 0.053*** | -0.019 | 0.054*** | -0.015 | -0.095*** | 0.028 | -0.001 | 0.050*** | -0.013 | -0.058*** |
|  | (0.015) | (0.018) | (0.014) | (0.016) | (0.016) | (0.015) | (0.019) | (0.015) | (0.013) | (0.015) |
| Age | -0.240*** | 0.146*** | 0.046*** | 0.052*** | 0.123*** | -0.268*** | 0.101*** | 0.047*** | 0.073*** | 0.134*** |
|  | (0.014) | (0.017) | (0.013) | (0.015) | (0.015) | (0.013) | (0.018) | (0.014) | (0.012) | (0.014) |
| Age squared | 0.003*** | -0.001*** | -0.001*** | -0.001*** | -0.001*** | 0.003*** | -0.001*** | -0.001*** | -0.001*** | -0.002*** |
|  | (0.000) | (0.000) | (0.000) | (0.000) | (0.000) | (0.000) | (0.000) | (0.000) | (0.000) | (0.000) |
| PC1 | -15.256*** | -2.295 | -4.115* | 5.472* | 18.520*** | -10.155*** | -4.785 | 0.030 | 0.176 | 13.827*** |
|  | (2.151) | (2.428) | (1.976) | (2.167) | (2.154) | (2.100) | (2.729) | (2.106) | (1.761) | (2.026) |
| PC2 | -2.341 | -1.834 | 0.778 | 1.306 | 0.440 | 3.357 | 1.120 | 2.472 | -2.110 | -3.296 |
|  | (2.438) | (2.670) | (1.954) | (2.061) | (2.066) | (2.400) | (2.664) | (2.259) | (1.810) | (2.025) |
| PC3 | 4.113 | -0.366 | -2.736 | -0.072 | -0.625 | 3.375 | -4.459 | 1.376 | 0.725 | -3.624 |
|  | (2.154) | (2.459) | (1.952) | (2.093) | (2.027) | (2.041) | (2.728) | (2.194) | (1.703) | (1.919) |
| Birth cohort dummies | Yes | Yes | Yes | Yes | Yes | Yes | Yes | Yes | Yes | Yes |
| Year dummies | Yes | Yes | Yes | Yes | Yes | Yes | Yes | Yes | Yes | Yes |
|  |  |  |  |  |  |  |  |  |  |  |
| Constant | 3.399*** | -4.560*** | -0.894* | -1.438*** | -3.572*** | 3.780*** | -3.537*** | -1.823*** | -1.254*** | -3.607*** |
|  | (0.354) | (0.426) | (0.332) | (0.367) | (0.366) | (0.329) | (0.458) | (0.348) | (0.287) | (0.344) |
|  |  |  |  |  |  |  |  |  |  |  |
| Pseudo-R^2^ | 0.130 | 0.019 | 0.027 | 0.021 | 0.037 | 0.129 | 0.015 | 0.012 | 0.024 | 0.041 |
| p(χ^2^) | *** | *** | *** | *** | *** | *** | *** | *** | *** | *** |

** P < 0.05, ** P < 0.005, *** P < 0.001; robust SE in parentheses; ADHD-PGI = Attention-Deficit/Hyperactivity Disorder polygenic index [11].*

**Supplemental Table 6.** Differences in predicted probabilities of each labour market status across ADHD-PGI quintiles relative to the lowest quintile

| **Sample** | **All** | **Males** | **Females** | **All with higher education** | **Males with higher education** | **Females with higher education** |
| --- | --- | --- | --- | --- | --- | --- |
| N | 19,707 | 9,068 | 10,639 | 7,456 | 2,998 | 4,458 |
| Average marginal effects: |  |  |  |  |  |  |
| **ADHD-PGI Q1** | Reference | Reference | Reference | Reference | Reference | Reference |
| **ADHD-PGI Q2** |  |  |  |  |  |  |
| Non-employed | 0.010 | 0.008 | 0.012 | 0.010 | -0.004 | 0.020 |
|  | (0.009) | (0.013) | (0.012) | (0.012) | (0.019) | (0.015) |
| Physical work | 0.009 | 0.000 | 0.016 | -0.004 | -0.016 | 0.003 |
|  | (0.009) | (0.014) | (0.011) | (0.007) | (0.011) | (0.009) |
| Knowledge work | -0.024* | -0.026* | -0.021 | -0.018 | -0.014 | -0.021 |
|  | (0.009) | (0.013) | (0.012) | (0.017) | (0.027) | (0.022) |
| **ADHD-PGI Q3** |  |  |  |  |  |  |
| Non-employed | 0.016 | 0.017 | 0.015 | 0.000 | -0.013 | 0.009 |
|  | (0.009) | (0.013) | (0.012) | (0.012) | (0.019) | (0.015) |
| Physical work | 0.024* | 0.033* | 0.017 | -0.000 | 0.003 | -0.003 |
|  | (0.009) | (0.014) | (0.011) | (0.007) | (0.012) | (0.009) |
| Knowledge work | -0.019* | -0.034* | -0.007 | 0.009 | -0.023 | 0.032 |
|  | (0.009) | (0.013) | (0.012) | (0.017) | (0.027) | (0.022) |
| **ADHD-PGI Q4** |  |  |  |  |  |  |
| Non-employed | 0.025* | 0.034* | 0.018 | 0.017 | 0.022 | 0.016 |
|  | (0.009) | (0.013) | (0.012) | (0.012) | (0.020) | (0.016) |
| Physical work | 0.041*** | 0.061*** | 0.023* | -0.002 | -0.002 | -0.003 |
|  | (0.009) | (0.015) | (0.011) | (0.007) | (0.012) | (0.009) |
| Knowledge work | -0.049*** | -0.067*** | -0.032* | -0.042* | -0.079** | -0.018 |
|  | (0.008) | (0.012) | (0.011) | (0.017) | (0.028) | (0.022) |
| **ADHD-PGI Q5** |  |  |  |  |  |  |
| Non-employed | 0.026** | 0.032* | 0.022 | 0.015 | 0.012 | 0.018 |
|  | (0.009) | (0.013) | (0.012) | (0.013) | (0.020) | (0.016) |
| Physical work | 0.040*** | 0.044** | 0.037*** | -0.002 | -0.005 | -0.001 |
|  | (0.009) | (0.014) | (0.011) | (0.007) | (0.012) | (0.009) |
| Knowledge work | -0.050*** | -0.063*** | -0.039*** | -0.030 | -0.052 | -0.016 |
|  | (0.008) | (0.013) | (0.011) | (0.018) | (0.029) | (0.022) |

** P < 0.05, ** P < 0.005, *** P < 0.001; robust SE in parentheses; ADHD-PGI = Attention-Deficit/Hyperactivity Disorder polygenic index [11]. Average marginal effects from probit models of labour market status, with ADHD-PGI quintiles as the main explanatory variable. Models control for gender, age (linear and squared), birth cohort dummies, study year dummies, and the first three genetic principal components (PC1–PC3).*

**Supplemental Table 7.** Coefficient estimates from probit models of labour market status in the higher education subsample

| **Sample** | **All with higher education** | **All with higher education** | **All with higher education** | **All with higher education** | **All with higher education** |
| --- | --- | --- | --- | --- | --- |
| **Labour market status** | **Non-employed** | **Self-employed** | **Physical**  **work** | **Office**  **work** | **Knowledge work** |
| **ADHD-PGI specification** | **Linear** | **Linear** | **Linear** | **Linear** | **Linear** |
| N | 7,456 | 7,456 | 7,456 | 7,456 | 7,456 |
| Model | probit | probit | probit | probit | probit |
| **Dependent variable: belonging to the specific labour market status** | | | | | |
| Explanatory variables: | | | | | |
| ADHD-PGI | 0.034 | 0.008 | -0.006 | 0.061* | -0.065** |
|  | (0.029) | (0.034) | (0.040) | (0.025) | (0.023) |
| Female | -0.053 | -0.251*** | -0.162** | 0.420*** | -0.246*** |
|  | (0.038) | (0.046) | (0.054) | (0.032) | (0.031) |
| ADHD-PGI # Female | -0.017 | 0.018 | 0.005 | -0.057 | 0.044 |
|  | (0.038) | (0.048) | (0.054) | (0.032) | (0.031) |
| Age | -0.300*** | 0.123*** | -0.050* | 0.033* | 0.162*** |
|  | (0.017) | (0.024) | (0.025) | (0.014) | (0.014) |
| Age squared | 0.004*** | -0.001*** | 0.001* | -0.001*** | -0.002*** |
|  | (0.000) | (0.000) | (0.000) | (0.000) | (0.000) |
| PC1 | -7.542** | 3.175 | -1.849 | -5.896* | 9.664*** |
|  | (2.664) | (3.079) | (3.639) | (2.120) | (2.056) |
| PC2 | 1.359 | -1.094 | 1.777 | -1.049 | 0.124 |
|  | (2.828) | (2.997) | (3.605) | (2.128) | (2.064) |
| PC3 | 0.358 | -1.137 | -0.479 | 3.966 | -3.129 |
|  | (2.608) | (3.007) | (3.963) | (2.070) | (2.031) |
| Birth cohort dummies | Yes | Yes | Yes | Yes | Yes |
| Year dummies | Yes | Yes | Yes | Yes | Yes |
|  |  |  |  |  |  |
| Constant | 4.404*** | -4.082*** | -0.676 | -0.851* | -3.477*** |
|  | (0.424) | (0.584) | (0.590) | (0.349) | (0.351) |
|  |  |  |  |  |  |
| Pseudo-R^2^ | 0.109 | 0.035 | 0.035 | 0.034 | 0.030 |
| p(χ^2^) | *** | *** | *** | *** | *** |

** P < 0.05, ** P < 0.005, *** P < 0.001; robust SE in parentheses; ADHD-PGI = Attention-Deficit/Hyperactivity Disorder polygenic index [11].*

**Supplemental Table 8.** Coefficient estimates from probit models of labour market status in the higher education subsample by gender

| **Sample** | **Males with higher education** | **Males with higher education** | **Males with higher education** | **Males with higher education** | **Males with higher education** | **Females with higher education** | **Females with higher education** | **Females with higher education** | **Females with higher education** | **Females with higher education** |
| --- | --- | --- | --- | --- | --- | --- | --- | --- | --- | --- |
| **Labour market status** | **Non-employed** | **Self-employed** | **Physical**  **work** | **Office**  **work** | **Knowledge work** | **Non-employed** | **Self-employed** | **Physical**  **work** | **Office**  **work** | **Knowledge work** |
| **ADHD-PGI specification** | **Linear** | **Linear** | **Linear** | **Linear** | **Linear** | **Linear** | **Linear** | **Linear** | **Linear** | **Linear** |
| N | 2,998 | 2,998 | 2,998 | 2,998 | 2,998 | 10,639 | 10,639 | 10,639 | 10,639 | 10,639 |
| Model | probit | probit | probit | probit | probit | probit | probit | probit | probit | probit |
| **Dependent variable: belonging to the specific labour market status** | | | | | | | | | | |
| Explanatory variables: | | | | | | | | | | |
| ADHD-PGI | 0.033 | 0.011 | -0.007 | 0.063* | -0.067** | 0.017 | 0.025 | -0.005 | 0.004 | -0.020 |
|  | (0.029) | (0.035) | (0.040) | (0.025) | (0.023) | (0.025) | (0.033) | (0.037) | (0.019) | (0.020) |
| Age | -0.279*** | 0.181*** | -0.070 | 0.018 | 0.146*** | -0.316*** | 0.087* | -0.025 | 0.036* | 0.172*** |
|  | (0.028) | (0.037) | (0.038) | (0.025) | (0.023) | (0.022) | (0.032) | (0.034) | (0.018) | (0.019) |
| Age squared | 0.003*** | -0.002*** | 0.001 | -0.000 | -0.002*** | 0.004*** | -0.001** | 0.000 | -0.001* | -0.002*** |
|  | (0.000) | (0.000) | (0.000) | (0.000) | (0.000) | (0.000) | (0.000) | (0.000) | (0.000) | (0.000) |
| PC1 | -7.305 | 2.717 | -4.365 | -6.150 | 9.253** | -7.643* | 3.859 | -0.793 | -5.871* | 9.760*** |
|  | (4.064) | (4.542) | (5.612) | (3.599) | (3.255) | (3.551) | (4.224) | (4.685) | (2.632) | (2.662) |
| PC2 | 1.963 | -7.093 | -7.446 | 0.403 | 2.595 | 0.965 | 5.369 | 8.989 | -2.122 | -1.841 |
|  | (3.601) | (5.361) | (5.464) | (3.382) | (3.096) | (4.268) | (3.988) | (5.012) | (2.737) | (2.807) |
| PC3 | 0.073 | -0.976 | -3.902 | 4.853 | -2.036 | 0.755 | -1.868 | 1.839 | 3.626 | -3.808 |
|  | (3.945) | (4.561) | (5.949) | (3.428) | (3.151) | (3.514) | (4.105) | (5.317) | (2.611) | (2.670) |
| Birth cohort dummies | Yes | Yes | Yes | Yes | Yes | Yes | Yes | Yes | Yes | Yes |
| Year dummies | Yes | Yes | Yes | Yes | Yes | Yes | Yes | Yes | Yes | Yes |
|  |  |  |  |  |  |  |  |  |  |  |
| Constant | 4.056*** | -5.744*** | -0.043 | -0.469 | -3.051*** | 4.569*** | -3.214*** | -1.537 | -0.531 | -4.001*** |
|  | (0.711) | (0.908) | (0.912) | (0.602) | (0.563) | (0.532) | (0.771) | (0.786) | (0.429) | (0.452) |
|  |  |  |  |  |  |  |  |  |  |  |
| Pseudo-R^2^ | 0.115 | 0.038 | 0.031 | 0.022 | 0.023 | 0.105 | 0.025 | 0.037 | 0.010 | 0.025 |
| p(χ^2^) | *** | *** | *** | *** | *** | *** | *** | *** | *** | *** |

** P < 0.05, ** P < 0.005, *** P < 0.001; robust SE in parentheses; ADHD-PGI = Attention-Deficit/Hyperactivity Disorder polygenic index [11].* **Supplemental Table 9.** Coefficient estimates from ordered probit models of equivalent income

| **Sample** | **All** | **Males** | **Females** | **All** | **Males** | **Females** |
| --- | --- | --- | --- | --- | --- | --- |
| **ADHD-PGI specification** | **Linear** | **Linear** | **Linear** | **Linear + squared** | **Linear + squared** | **Linear + squared** |
| N | 19,685 | 9,098 | 10,587 | 19,685 | 9,098 | 10,587 |
| Model | oprobit | oprobit | oprobit | oprobit | oprobit | oprobit |
| **Dependent variable: Equivalent income** | | | | | | |
| Explanatory variables: | | | | | | |
| ADHD-PGI | -0.035** | -0.034** | -0.031** | -0.033*** | -0.034** | -0.031** |
|  | (0.012) | (0.012) | (0.011) | (0.008) | (0.012) | (0.011) |
| ADHD-PGI squared |  |  |  | -0.008 | -0.009 | -0.005 |
|  |  |  |  | (0.006) | (0.008) | (0.008) |
| Female | -0.088*** |  |  | -0.088*** |  |  |
|  | (0.016) |  |  | (0.016) |  |  |
| ADHD-PGI # Female | 0.005 |  |  |  |  |  |
|  | (0.016) |  |  |  |  |  |
| Age | 0.111*** | 0.120*** | 0.104*** | 0.111*** | 0.120*** | 0.104*** |
|  | (0.007) | (0.011) | (0.010) | (0.007) | (0.011) | (0.010) |
| Age squared | -0.001*** | -0.001*** | -0.001*** | -0.001*** | -0.001*** | -0.001*** |
|  | (0.000) | (0.000) | (0.000) | (0.000) | (0.000) | (0.000) |
| PC1 | 13.490*** | 15.650*** | 11.374*** | 13.509*** | 15.680*** | 11.385*** |
|  | (1.150) | (1.684) | (1.580) | (1.150) | (1.684) | (1.580) |
| PC2 | -2.237 | -2.023 | -2.388 | -2.213 | -1.957 | -2.388 |
|  | (1.238) | (1.865) | (1.671) | (1.238) | (1.865) | (1.670) |
| PC3 | -3.816*** | -4.411* | -3.386* | -3.813*** | -4.403* | -3.379* |
|  | (1.125) | (1.676) | (1.523) | (1.125) | (1.676) | (1.523) |
| Birth cohort dummies | Yes | Yes | Yes | Yes | Yes | Yes |
| Year dummies | Yes | Yes | Yes | Yes | Yes | Yes |
|  |  |  |  |  |  |  |
| Cut: Lowest/Medium equivalent income | 1.709*** | 2.017*** | 1.555*** | 1.703*** | 2.009*** | 1.551*** |
|  | (0.184) | (0.272) | (0.250) | (0.184) | (0.272) | (0.250) |
| Cut: Medium/Highest equivalent income | 2.642*** | 2.914*** | 2.521*** | 2.636*** | 2.906*** | 2.516*** |
|  | (0.184) | (0.273) | (0.250) | (0.184) | (0.273) | (0.250) |
|  |  |  |  |  |  |  |
| Pseudo-R^2^ | 0.022 | 0.021 | 0.021 | 0.022 | 0.022 | 0.021 |
| p(χ^2^) | *** | *** | *** | *** | *** | *** |

** P < 0.05, ** P < 0.005, *** P < 0.001; robust SE in parentheses; ADHD-PGI = Attention-Deficit/Hyperactivity Disorder polygenic index [11].*

**Supplemental Table 10.** Differences in predicted probabilities of belonging to each equivalent income tertile across ADHD-PGI quintiles relative to the lowest quintile

| **Sample** | **All** | **Males** | **Females** |
| --- | --- | --- | --- |
| N | 19,685 | 9,098 | 10,587 |
| Average marginal effects: |  |  |  |
| **ADHD-PGI Q1** | Reference | Reference | Reference |
| **ADHD-PGI Q2** |  |  |  |
| Lowest equivalent income | 0.008 | 0.012 | 0.005 |
|  | (0.009) | (0.013) | (0.012) |
| Medium equivalent income | 0.000 | 0.001 | -0.000 |
|  | (0.000) | (0.001) | (0.000) |
| Highest equivalent income | -0.008 | -0.012 | -0.005 |
|  | (0.009) | (0.013) | (0.012) |
| **ADHD-PGI Q3** |  |  |  |
| Lowest equivalent income | 0.001 | 0.005 | -0.002 |
|  | (0.009) | (0.013) | (0.012) |
| Medium equivalent income | 0.000 | 0.000 | 0.000 |
|  | (0.000) | (0.001) | (0.000) |
| Highest equivalent income | -0.001 | -0.005 | 0.002 |
|  | (0.009) | (0.013) | (0.012) |
| **ADHD-PGI Q4** |  |  |  |
| Lowest equivalent income | 0.030*** | 0.023 | 0.037** |
|  | (0.009) | (0.013) | (0.013) |
| Medium equivalent income | -0.001 | 0.001 | -0.003* |
|  | (0.000) | (0.001) | (0.001) |
| Highest equivalent income | -0.030*** | -0.023 | -0.034** |
|  | (0.009) | (0.013) | (0.012) |
|  |  |  |  |
| **ADHD-PGI Q5** |  |  |  |
| Lowest equivalent income | 0.028** | 0.033* | 0.024 |
|  | (0.009) | (0.013) | (0.012) |
| Medium equivalent income | -0.000 | 0.001 | -0.001 |
|  | (0.000) | (0.001) | (0.001) |
| Highest equivalent income | -0.028** | -0.033* | -0.023 |
|  | (0.009) | (0.013) | (0.012) |

** P < 0.05, ** P < 0.005, *** P < 0.001; robust SE in parentheses; ADHD-PGI = Attention-Deficit/Hyperactivity Disorder polygenic index [11]. Average marginal effects from ordered probit models of equivalent income, with ADHD-PGI quintiles as the main explanatory variable. Models control for gender, age (linear and squared), birth cohort dummies, study year dummies, and the first three genetic principal components (PC1–PC3).*

**Supplemental Table 11.** Coefficient estimates from ordered probit models of equivalent income in educational and occupational categories

| **Sample** | **All with higher education** | **Males with higher education** | **Females with higher education** | **All in physical work** | **All in office work** | **All in knowledge work** |
| --- | --- | --- | --- | --- | --- | --- |
| **ADHD-PGI specification** | **Linear** | **Linear** | **Linear** | **Linear** | **Linear** | **Linear** |
| N | 19,685 | 9,098 | 10,587 | 4,065 | 5,429 | 3,382 |
| Model | oprobit | oprobit | oprobit | oprobit | oprobit | oprobit |
| **Dependent variable: Equivalent income** | | | | | | |
| Explanatory variables: | | | | | | |
| ADHD-PGI | -0.059* | -0.061** | -0.020 | -0.015 | 0.005 | -0.054 |
|  | (0.022) | (0.021) | (0.018) | (0.023) | (0.030) | (0.032) |
| Female | -0.167*** |  |  | -0.427*** | -0.280*** | -0.201*** |
|  | (0.028) |  |  | (0.037) | (0.035) | (0.043) |
| ADHD-PGI # Female | 0.038 |  |  | 0.051 | -0.039 | 0.061 |
|  | (0.028) |  |  | (0.036) | (0.035) | (0.044) |
| Age | 0.105*** | 0.128*** | 0.097*** | 0.047* | 0.057*** | 0.030 |
|  | (0.013) | (0.021) | (0.016) | (0.017) | (0.015) | (0.023) |
| Age squared | -0.001*** | -0.001*** | -0.001*** | -0.000* | -0.001*** | 0.000 |
|  | (0.000) | (0.000) | (0.000) | (0.000) | (0.000) | (0.000) |
| PC1 | 8.185*** | 7.724* | 8.482*** | 9.432*** | 6.244** | 9.781*** |
|  | (1.858) | (3.000) | (2.376) | (2.596) | (2.224) | (2.925) |
| PC2 | -2.231 | -0.952 | -2.961 | -2.768 | -1.017 | -2.529 |
|  | (1.945) | (2.997) | (2.611) | (2.919) | (2.489) | (2.634) |
| PC3 | -2.830 | -3.554 | -2.538 | -2.768 | -3.212 | -1.556 |
|  | (1.852) | (3.034) | (2.351) | (2.569) | (2.134) | (2.863) |
| Birth cohort dummies | Yes | Yes | Yes | Yes | Yes | Yes |
| Year dummies | Yes | Yes | Yes | Yes | Yes | Yes |
|  |  |  |  |  |  |  |
| Cut: Lowest/Medium equivalent income | 1.314*** | 2.026*** | 1.189** | 0.493 | 0.189 | -0.306 |
|  | (0.304) | (0.510) | (0.379) | (0.404) | (0.356) | (0.548) |
| Cut: Medium/Highest equivalent income | 2.286*** | 2.960*** | 2.187*** | 1.737*** | 1.391*** | 0.724 |
|  | (0.305) | (0.511) | (0.380) | (0.404) | (0.357) | (0.547) |
|  |  |  |  |  |  |  |
| Pseudo-R^2^ | 0.032 | 0.032 | 0.028 | 0.034 | 0.031 | 0.051 |
| p(χ^2^) | *** | *** | *** | *** | *** | *** |

** P < 0.05, ** P < 0.005, *** P < 0.001; robust SE in parentheses; ADHD-PGI = Attention-Deficit/Hyperactivity Disorder polygenic index [11].*

**Supplemental Table 12.** Coefficient estimates from probit models of economic satisfaction

| **Sample** | **All** | **Males** | **Females** | **All** | **Males** | **Females** | **All in lowest equivalent income tertile** | **Males in lowest equivalent income tertile** | **Females in lowest equivalent income tertile** |
| --- | --- | --- | --- | --- | --- | --- | --- | --- | --- |
| **ADHD-PGI specification** | **Linear** | **Linear** | **Linear** | **Linear + squared** | **Linear + squared** | **Linear + squared** | **Linear** | **Linear** | **Linear** |
| N | 20,029 | 9,201 | 10,828 | 20,029 | 9,201 | 10,828 | 6,489 | 2,881 | 3,608 |
| Model | probit | probit | probit | probit | probit | probit | probit | probit | probit |
| **Dependent variable: Economic satisfaction** | | | | | | | | | |
| Explanatory variables: | | | | | | | | | |
| ADHD-PGI | -0.065*** | -0.065*** | -0.038* | -0.051*** | -0.064*** | -0.038* | -0.092*** | -0.095*** | -0.015 |
|  | (0.015) | (0.015) | (0.015) | (0.011) | (0.015) | (0.015) | (0.025) | (0.025) | (0.023) |
| ADHD-PGI squared |  |  |  | -0.004 | -0.008 | -0.000 |  |  |  |
|  |  |  |  | (0.008) | (0.011) | (0.010) |  |  |  |
| Female | 0.073*** |  |  | 0.074*** |  |  | 0.120*** |  |  |
|  | (0.021) |  |  | (0.021) |  |  | (0.033) |  |  |
| ADHD-PGI # Female | 0.026 |  |  |  |  |  | 0.076* |  |  |
|  | (0.021) |  |  |  |  |  | (0.033) |  |  |
| Age | 0.021* | 0.024 | 0.019 | 0.021* | 0.024 | 0.019 | -0.025 | -0.029 | -0.019 |
|  | (0.010) | (0.014) | (0.014) | (0.010) | (0.014) | (0.014) | (0.015) | (0.022) | (0.020) |
| Age squared | -0.000 | -0.000 | -0.000 | -0.000 | -0.000 | -0.000 | 0.000* | 0.000 | 0.000 |
|  | (0.000) | (0.000) | (0.000) | (0.000) | (0.000) | (0.000) | (0.000) | (0.000) | (0.000) |
| PC1 | -3.831* | -2.911 | -4.730* | -3.817* | -2.871 | -4.730* | -7.458*** | -8.671* | -6.280* |
|  | (1.465) | (2.124) | (2.022) | (1.464) | (2.123) | (2.022) | (2.239) | (3.296) | (3.066) |
| PC2 | -3.591* | -1.867 | -5.160* | -3.588* | -1.805 | -5.160* | -0.417 | 1.251 | -1.936 |
|  | (1.452) | (2.137) | (2.046) | (1.452) | (2.136) | (2.045) | (2.081) | (2.887) | (3.008) |
| PC3 | 3.128* | 1.919 | 4.210* | 3.100* | 1.931 | 4.210* | 7.288*** | 6.943* | 7.885* |
|  | (1.469) | (2.125) | (2.049) | (1.469) | (2.125) | (2.049) | (2.104) | (3.025) | (2.954) |
| Birth cohort dummies | Yes | Yes | Yes | Yes | Yes | Yes | Yes | Yes | Yes |
| Year dummies | Yes | Yes | Yes | Yes | Yes | Yes | Yes | Yes | Yes |
| Constant | 0.181 | 0.081 | 0.356 | 0.183 | 0.089 | 0.356 | 0.695 | 0.783 | 0.700 |
|  | (0.242) | (0.354) | (0.332) | (0.242) | (0.354) | (0.332) | (0.366) | (0.550) | (0.491) |
|  |  |  |  |  |  |  |  |  |  |
| Pseudo-R^2^ | 0.019 | 0.021 | 0.022 | 0.019 | 0.021 | 0.022 | 0.034 | 0.037 | 0.034 |
| p(χ^2^) | *** | *** | *** | *** | *** | *** | *** | *** | *** |

** P < 0.05, ** P < 0.005, *** P < 0.001; robust SE in parentheses; ADHD-PGI = Attention-Deficit/Hyperactivity Disorder polygenic index [11].*

**Supplemental Table 13.** Differences in predicted probabilities of economic satisfaction across ADHD-PGI quintiles relative to the lowest quintile

| **Sample** | **All** | **Males** | **Females** | **All in lowest equivalent income tertile** | **Males in lowest equivalent income tertile** | **Females in lowest equivalent income tertile** |
| --- | --- | --- | --- | --- | --- | --- |
| N | 20,029 | 9,201 | 10,828 | 6,489 | 2,881 | 3,608 |
| Average marginal effects: |  |  |  |  |  |  |
| **ADHD-PGI Q1** | Reference | Reference | Reference | Reference | Reference | Reference |
| **ADHD-PGI Q2** | -0.017* | -0.015 | -0.019 | -0.020 | -0.018 | -0.026 |
|  | (0.008) | (0.012) | (0.011) | (0.018) | (0.027) | (0.024) |
| **ADHD-PGI Q3** | -0.023* | -0.029* | -0.016 | -0.036* | -0.049 | -0.025 |
|  | (0.008) | (0.012) | (0.011) | (0.018) | (0.027) | (0.024) |
| **ADHD-PGI Q4** | -0.039*** | -0.047*** | -0.032** | -0.042* | -0.088** | -0.010 |
|  | (0.008) | (0.012) | (0.011) | (0.018) | (0.027) | (0.023) |
| **ADHD-PGI Q5** | -0.038*** | -0.046*** | -0.031* | -0.056** | -0.089*** | -0.031 |
|  | (0.008) | (0.012) | (0.011) | (0.018) | (0.027) | (0.024) |

** P < 0.05, ** P < 0.005, *** P < 0.001; robust SE in parentheses; ADHD-PGI = Attention-Deficit/Hyperactivity Disorder polygenic index [11]. Average marginal effects from probit models of economic satisfaction, with ADHD-PGI quintiles as the main explanatory variable. Models control for gender, age (linear and squared), birth cohort dummies, study year dummies, and the first three genetic principal components (PC1–PC3).*
